# Targeting Autophagy to Overcome Chemoresistance and Immune Resistance in Triple-Negative Breast Cancer

**DOI:** 10.3390/cancers18091359

**Published:** 2026-04-24

**Authors:** Shubham D. Mishra, Patricia Mendonca, Sukhmandeep Kaur, Karam F. A. Soliman

**Affiliations:** 1 Division of Pharmaceutical Sciences, College of Pharmacy and Pharmaceutical Sciences, Institute of Public Health, Florida A&M University, Tallahassee, FL 32307, USA; shubham1.mishra@famu.edu (S.D.M.); sukhmandeep1.kaur@famu.edu (S.K.); 2 Department of Biology, College of Science and Technology, Florida A&M University, Tallahassee, FL 32307, USA

**Keywords:** autophagy, chemoresistance, immune resistance, TNBC, tumor microenvironment, immunotherapy, PI3K/AKT/mTOR, cGAS-STING signaling, cancer stem cells (CSCs), immune checkpoint blockade

## Abstract

Triple-negative breast cancer (TNBC) remains one of the most challenging subtypes of breast cancer to treat, defined by its molecular heterogeneity, absence of hormone receptors, and poor clinical outcomes. While this difficulty with cancer cells persists even in the presence of chemotherapy and immune checkpoint inhibitors (ICIs), one critical factor linked to both chemoresistance and immune escape is autophagy. Autophagy is a cellular process with lysosomal recycling function. In TNBC, autophagy paradoxically shifts from tumor-suppressive to a tumor-promoting role. Autophagy was initially known to maintain genomic stability and alleviate oxidative damage. In TNBC, cancer cells use autophagy to detoxify platinum-induced DNA. damage, clear damaged mitochondria via mitophagy, recycle critical macromolecules, and sustain dormancy in cancer stem-like cells (CSCs). At the same time, the process of autophagic flux facilitates immune evasion, including PD-L1 expression stabilization, MHC-I degradation, and the establishment of an immunosuppressive tumor microenvironment (TME). The review encapsulates the progressive concepts of molecular regulation of autophagy, which involve key factors such as ULK1, VPS34, and non-coding RNAs (ncRNAs). These factors play a significant role in chemoresistance, taxanes, anthracyclines, and platinum compounds. The review also discusses various strategies for translation that aim to circumvent or suppress autophagy-mediated chemoresistance, including autophagy inhibitors, natural compounds, and nanoparticle-based formulations, with a focus on their synergistic potential with ICIs and chemotherapeutic agents. Targeting autophagy has shown considerable potential for effectively addressing chemoresistance in TNBC. Future studies should focus on addressing chemoresistance and immunoresistance through autophagy-based therapies.

## Simple Summary

Triple-negative breast cancer (TNBC) is among the most challenging subtypes of breast cancer. Although advances in chemotherapy and emerging immune-based therapies have improved options, many TNBC patients develop resistance after treatment. Autophagy-a natural cellular recycling process essential for maintaining homeostasis of the cancer cells to repair treatment-induced damage, evade cell death, and escape immune surveillance. This review examines how autophagy is dysregulated in TNBC, the key molecular pathways, and how this altered process contributes to both chemotherapy and immunotherapy. The review also highlights emerging therapeutic strategies that aim to inhibit or reprogram autophagy to improve treatment response and overcome resistance in TNBC.

## 1. Introduction

Treatment of triple-negative breast cancer (TNBC) remains one of the most challenging conditions due to the absence of estrogen receptor (ER), progesterone receptor (PR), and HER2, and the inability to respond to conventionally targeted therapies. TNBC represents approximately 15–20% of all breast cancers, but also represents a larger percentage of associated mortality, approximately 30–40%. TNBC exhibits aggressive clinical behavior, with nearly half of patients experiencing recurrence within three years. It also tends to spread more quickly to vital organs such as the brain, lungs, and liver, and in more advanced cases, the median survival time drops to less than 18 months [1,2].

Current treatment approaches for TNBC include standard chemotherapy with agents such as anthracyclines, taxanes, and platinum agents. Newer treatments include immune checkpoint inhibitors (ICIs) for PD-L1-positive tumors, PARP inhibitors for tumors with BRCA mutations, and sacituzumab govitecan. However, these treatments often show limited success, and drug resistance is a frequent problem. This is likely because there are limited broad-spectrum molecular targets [3,4]. In particular, the lack of specific molecular targets limits the utility of cytotoxic chemotherapy with anthracyclines, taxanes, and platinum agents in first-line TNBC therapy. Also, more than 70% of patients develop resistance to chemotherapy over time, which is caused by increased activity of certain transport proteins called ATP-binding cassette (ABC) transporters, improved ways the cancer cells repair DNA damage, and changes in the tumor environment itself [3,5].

Beyond chemotherapy, immunotherapy has also emerged as an exciting option for treating TNBC. Clinical studies evaluating immunotherapy—including KEYNOTE-522 and IMpassion130—have shown benefit to the addition of the ICIs to chemotherapy, with favorable results such as pathologic response and progression-free survival; however, resistance remains a significant limitation [6,7,8]. Development of resistance involves multiple molecular pathways and cellular mechanisms. These include myeloid cells that inhibit immune response, T-cell exhaustion, and decreased or fluctuating PD-L1 levels [4,9,10]. Despite the presence of PD-L1+ cells, about 60% of tumors exhibit primary resistance to ICIs such as pembrolizumab. This resistance mainly stems from T-cell exhaustion, the accumulation of myeloid-derived suppressor cells (MDSCs), and cytokine-driven immunosuppression [6]. The dual resistance crisis and TNBC genomic heterogeneity at basal-like, mesenchymal, and immunomodulatory subtypes necessitate targeting strategies against universal survival pathways that facilitate cellular adaptation and immune evasion [11].

The autophagy pathway, which involves lysosomal degradation, is also a master regulator of TNBC’s therapeutic resistance. Autophagy is controlled by the ULK1 complex (Unc-51-like kinase 1), Beclin-1/VPS34-dependent nucleation, LC3 (microtubule-associated protein 1 light chain 3) lipidation, and SNARE-mediated fusion that maintains homeostasis under stress conditions by engulfing damaged cargo into autophagosomes for lysosomal destruction [12]. While autophagy suppresses tumorigenesis by preventing genomic instability, TNBC tumors also exploit it as a cytoprotective defense [13]. Electron microscopic examination of autophagy for cancer cells has reported high numbers of autophagosomes in chemoresistant TNBC models and, therefore, linked autophagy to the survival of cancer cells under cytotoxic stress [14,15]. As a platinum-based chemotherapeutic, cisplatin produces DNA crosslinks that inhibit both transcription and replication, and has shown clinical benefit in TNBC, with a specific benefit observed in TNBC with defects in DNA repair. Unfortunately, in addition to the standard therapeutic effects of cisplatin, exposure also increases autophagic flux, which TNBC cells use for survival. Augmented autophagy facilitates the clearance of drug-DNA adducts, the recycling of macromolecules to produce ATP/nucleotides, and the degradation of pro-apoptotic proteins, thereby contributing to chemoresistance [16]. In line with mechanistic observations, higher LC3B expression is associated with a poor prognosis and increased chemoresistance in TNBC, demonstrating the functional relevance of LC3B in a pathological context [17]. At the same time, autophagy promotes immune evasion, in which p62/NF-κB signals stabilize PD-L1 mRNA, helping immune checkpoints evade detection. In addition, the secretion of TGF-β also attracts regulatory T cells (Tregs) and myeloid-derived suppressor cells (MDSCs), while the degradation of major histocompatibility complex class I (MHC-I) also disrupts antigen presentation [9,10]. The PI3K/AKT/mTOR pathway, which is frequently present in TNBC, is often deregulated. During prolonged activation of the AKT pathway, mTOR-mediated feedback inhibition can surprisingly trigger autophagy, leading to treatment resistance [18,19]. Breast cancer cells have been shown to rapidly adapt by activating autophagy pathways that help detoxify platinum-DNA adducts [20,21]. Preclinical studies have shown that hydroxychloroquine (HCQ), a lysosomal inhibitor, may be effective in overcoming platinum resistance by inhibiting autophagic flux. When combined with anti-PD-1 therapy, HCQ not only restored cisplatin sensitivity but also markedly increased CD8+ T-cell infiltration in TNBC models [22]. This is because inhibition of autophagy prevents the overdegradation of tumor antigens and MHC-I molecules, thereby promoting antigen presentation and restoration of immune desertification [9,10]. In cancer-rich TNBC cells, which act as a reservoir for tumor recurrence, selective autophagy helps them remain dormant and survive in low-oxygen environments, much as stem cells do [23,24,25].

This review explains how autophagy can confer dual resistance and examines its role in controlling chemoresistance to taxanes (by clearing tubulin aggregates), anthracyclines (through mitophagy), and platinum agents (by recycling nucleotides). At the same time, it helps tumors avoid immune detection by stabilizing PD-L1 and stopping T cell activity. The review also examines new treatments, such as ULK1 inhibitors, VPS34 inhibitors, and autophagy modifiers, delivered via nanoparticles with ICIs and chemotherapy [26,27]. By integrating molecular oncology with translational immunology, this review aims to establish autophagy inhibition as a unified strategy to inhibit TNBC’s dual resistance, ultimately fulfilling the promise of extending survival for patients burdened by this disease [28,29]. Although autophagy is a crucial cellular process observed in several types of cancer, TNBC exhibits unique features, including genomic instability, altered metabolism, and an immunosuppressive tumor microenvironment (TME).

## 2. Methods of Literature Review

To identify relevant research, a thorough literature search was conducted through PubMed, Scopus, and Web of Science, focusing on autophagy, immunotherapy, and treatment resistance in TNBCs. The articles that appeared in the time frame of 2000 to 2025 were selected. The keyword search terms included “autophagy,” “TNBC,” “immune checkpoint inhibitors,” “therapeutic resistance,” and “tumor microenvironment.” Sources included original research articles and reviews that emphasized recent, highly cited, and mechanistic papers. The exclusion criteria included studies that were not directly related to TNBC or did not involve autophagy-based mechanisms.

## 3. Overview of Triple-Negative Breast Cancer

TNBC is characterized by the lack of expression of ER, PR, and HER2, making it unresponsive to endocrine or HER2-targeted therapies. In 2025, the American Cancer Society estimates that there will be 316,950 new cases of invasive breast cancer in women in the U.S., and 10–15% (31,700–47,500 cases) will be TNBC [30,31]. Epidemiologic surveillance indicates that TNBC incidence has remained relatively stable over the last decade, even as overall breast cancer rates have trended upward in younger age groups. This epidemiological burden, along with the aggressive clinical behavior of TNBC and the absence of approved targeted therapies, emphasizes its significance as a critical public health issue [30,31].

TNBC is more commonly found in Hispanic and African American women and usually affects premenopausal women. Moreover, individuals who carry the germline BRCA1 mutation appear to be at higher risk. Foulkes et al. (2010) reported that BRCA1 mutation carriers are up to 10 times more likely to develop TNBC than the general population [32]. Bauer et al. (2007) reported that African American women were at nearly double the incidence of TNBC compared to Caucasian women [33]. Moreover, Carey et al. (2006) also examined disparities in survival; they concluded that African American patients with TNBC were 38% more likely to die than Caucasian patients, even after adjusting for stage, grade, and socioeconomic status [34]. In conclusion, social and genetic factors are important components of the epidemiology of TNBC, highlighting the possible need for different screening and prevention strategies for those at-risk individuals [32,33,34].

Clinically, TNBC presents as rapidly enlarging, high-grade invasive ductal carcinomas with infiltrating lymphocytes, central necrosis, and often has visceral or brain metastases at the time of recurrence [1,2]. Dent et al. (2007) documented that the median time to recurrence of some breast cancers is less than three years [2]. This is substantially different from the longer-term risks observed in hormone receptor-positive breast cancers. At diagnosis, TNBC can often present with both lymph node positivity and larger tumors, which compromise prognosis even further in the absence of targeted therapy. The aggressive histopathologic and clinical characteristics of TNBC necessitate timely diagnosis and initiation of systemic therapy to optimize outcomes [1,2].

Recent molecular profiling studies have revealed significant heterogeneity within TNBC. In this regard, Lehmann et al. (2011) have suggested that TNBC should be classified into six unique subtypes: basal-like 1, basal-like 2, immunomodulatory, mesenchymal, mesenchymal stem-like, and luminal androgen receptor [11]. Each subtype exhibits distinct gene expression patterns, pathway dependencies, and interactions with the microenvironment, leading to varying levels of chemosensitivity and immunogenicity. This profiling has facilitated the development of biomarker-based classification, including basal-like 2 and luminal AR subtypes, as well as drugs that block androgen receptors. Precision medicine for TNBC is advancing; however, the complexity of subtype classification and the dynamic nature of tumors still hinder its clinical application [11]

### 3.1. Triple-Negative Breast Cancer Therapeutic Targets

Targeted therapy in TNBC has focused on identifying key nodes and molecular mediators of resistance and progression. Activation of the PI3K/AKT/mTOR pathway is a common event, often secondary to PTEN deletion or PIK3CA mutation, leading to proliferation and survival via autophagy. Dual inhibition of PI3K or AKT, combined with mTOR inhibition, has been shown to reverse chemoresistance and promote apoptosis [29,35,36]. A potent strategy targeting the PI3K/AKT/mTOR pathway, in combination with autophagy inhibition, has been indicated to enhance metabolic resilience [37]. Targeting DNA repair iniparib targeting is another major strategy. PARP inhibitors such as olaparib and talazoparib target homologous recombination defects, exploiting synthetic lethality in BRCA1/2-mutant cancers [38,39]. Building on this concept, ATR and CHK1 inhibitors can complement PI3K/mTOR inhibition to induce genotoxic stress [37,40,41].

Overexpression of EGFR and activation of the AR signaling pathway are two additional potential targets. Monotherapy with cetuximab targeting EGFR, in combination with EGFR and PI3K inhibition, can inhibit compensatory activation [42]. AR antagonists such as enzalutamide inhibit proliferation in luminal-AR TNBC, and combination inhibition with mTOR has been shown to be synergistic [7,11,37]. Targeting angiogenesis and metabolism by inhibiting VEGF, as well as metabolic enzymes (such as LDHA, GLS), suppresses tumor angiogenesis and progression [43]. A strategy proposed by Xu et al. (2020) to overcome hypoxia-evoked Cadherin switching and subsequent autophagy/neovascularization escape is a combination [37].

Other promising approaches target cancer stem cell (CSC) pathways, including Notch and Wnt/β-catenin, which, when combined with inhibition of CSC properties, lead to increased chemosensitivity [44]. Epigenetic modifiers are also promising, including HDAC and BET, which, when combined with immunotherapy, enhance the immunogenicity of cancer [6,45]. In addition, biologically active agents, including curcumin and quercetin, when administered via nanoparticles, lead to multitargeted inhibition of PI3K/AKT, NF-κB, and MAPK pathways with minimal toxicity [37]. When used together, molecular inhibitors, natural agents, autophagy modifiers, and immune checkpoint therapies offer a wide range of treatment options for TNBC. Going forward, it is very important to focus on combination therapies based on biomarkers that target mechanisms of resistance that are similar [7,29,37].

### 3.2. Current Therapeutic Strategies and Emerging Challenges in TNBC

Anthracycline- and taxane-based neoadjuvant chemotherapy remains the foundation of early-stage TNBC treatment, achieving pathological complete response (pCR) rates of 30–40% [46]. Achieving pCR is linked to a 70% lower risk of recurrence and a 5-year event-free survival rate that is twice as high as that of patients with residual disease. Nonetheless, nearly 60% of patients exhibit an incomplete response, leading to early relapses. Strategies to intensify neoadjuvant regimens, such as the addition of platinum salts, have modestly improved pCR rates but at the cost of increased hematologic toxicity, underscoring the need for novel combinatorial approaches [46].

ICIs have redefined the treatment paradigm in TNBC. In the KEYNOTE-522 trial (a Phase III randomized, double-blind clinical trial in early-stage TNBC), the addition of pembrolizumab to neoadjuvant chemotherapy elevated pCR rates from 51.2% to 64.8% and improved event-free survival at two years [6]. In the IMpassion130 trial (a Phase III multicenter, randomized, double-blind, placebo-controlled trial in metastatic TNBC), first-line therapy with atezolizumab plus nab-paclitaxel in patients with PD-L1-positive metastatic TNBC extended median progression-free survival from 5.5 to 7.2 months and improved median overall survival from 15.5 to 25.0 months compared with chemotherapy alone [47]. Although improvements in overall survival remain modest, advances have made immunotherapy a vital element in the treatment of TNBC. Additionally, they support the continuous exploration of detecting predictive biomarkers and understanding the mechanisms of resistance [6,47].

However, despite these advances, resistance to immunotherapy remains a significant barrier. Patients with primary resistance have tumors that lack sufficient tumor-infiltrating lymphocytes (TILs) (Prognostic markers), have low PD-L1 expression, or harbor immune-suppressive myeloid populations, such as tumor-associated macrophages and myeloid-derived suppressor cells [4,9,48,49,50]. These findings suggest that ICIs exhibit limited efficacy in this tumor subset. Tumors that were initially responsive can develop acquired resistance. This may result from processes such as T-cell exhaustion and the induction of alternative immune checkpoint pathways, disruption of MHC-I, which in turn prevents antigen presentation, and changes to the TME to an immune-excluded or immune-desert state [10,27,45,51,52,53]. These resistance mechanisms are why more than half of TNBC patients are unable to maintain responses to ICIs, and there is a critical need for rational combination therapeutic approaches that incorporate immunotherapy with agents targeting autophagy, the DNA damage response, or PI3K/AKT/mTOR signaling [6,22,35,36,40,47,54].

**Figure 1:**
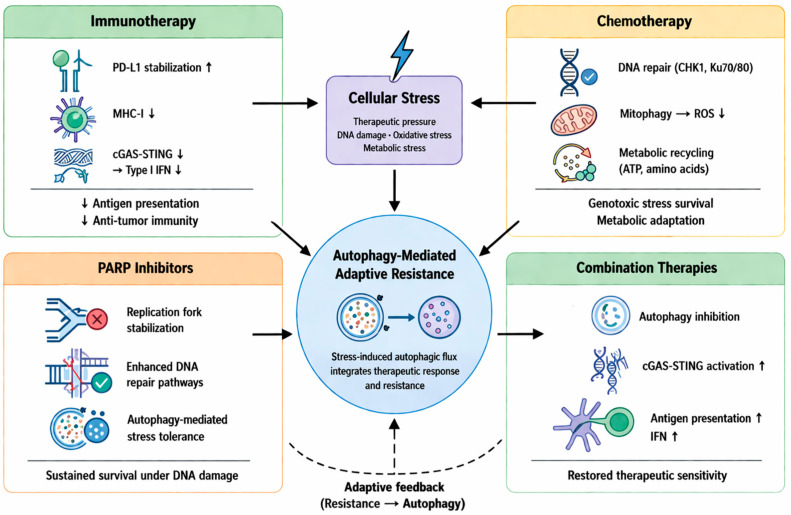
Adaptive resistance mediated by autophagy in TNBC. In this figure, one can see how immunotherapy, chemotherapy, and PARP inhibitors induce stress conditions (e.g., DNA damage, oxidative, and metabolic stress) in TNBC cells, thereby triggering the adaptive response of autophagy. Autophagy contributes to immune escape by stabilizing PD-L1, lowering MHC-I levels, and inhibiting the cGAS-STING pathway, thereby decreasing type I interferon production and antigen presentation. Moreover, chemotherapy-stimulated autophagy contributes to DNA repair (CHK1, Ku70/80), ROS degradation via mitophagy, and metabolic recycling to support cell survival under stress. Autophagy in response to PARP inhibitors contributes to resistance by stabilizing replication forks and repairing DNA. Generally, all these mechanisms result in autophagy-dependent adaptive resistance, which is further amplified by positive feedback from adaptive resistance to autophagy activation. On the other hand, combined treatments blocking autophagy promote cGAS-STING activity, antigen presentation, and interferon production. The upward (↑) and downward (↓) arrows show upregulation and downregulation, respectively. This schematic was created with the assistance of AI-based visualization tools.

In recent years, targeted therapies have begun to reshape the treatment landscape for select TNBC subgroups, complementing the gains achieved with immunotherapy. In germline BRCA-mutated metastatic TNBC, the PARP inhibitors olaparib and talazoparib have been shown to extend progression-free survival by approximately 3 months compared with conventional regimens [38,39]. Another notable advance is the antibody-drug conjugate sacituzumab govitecan, which delivers a cytotoxic payload directly to Trop-2-expressing tumor cells. In heavily pretreated patients, this therapy improved the median overall survival by 5.7 months compared with standard chemotherapy [55]. The recognition of HER2-low expression has now expanded the scope of trastuzumab deruxtecan to include patient populations previously considered HER2-negative. The response rates have exceeded 50% in clinical series. Together, these advances illustrate the potential of exploiting distinct molecular vulnerabilities in TNBC [43], including combination immunotherapies, such as bispecific antibodies, STING (Stimulator of Interferon Genes) pathway agonists, and, most interestingly, inhibitors of the PI3K/AKT/mTOR signaling cascade; all aimed at preventing both primary and acquired resistance in the experimental phase. Additionally, prognostic markers such as tumor-infiltrating lymphocyte density, which correlated with outcome and the probability of response to immunotherapy, are increasingly being integrated into the study design [4]. Synthetic lethal approaches targeting DNA damage response pathways, including ATR and CHK1 inhibitors, have shown promising preclinical activity in basal-like TNBC [40]. Moreover, combining liquid biopsy monitoring with adaptive clinical trial designs could streamline drug development, inform real-time treatment, and better select therapies in distinct TNBC subtypes (Figure 1) [4,40].

## 4. Autophagy: Molecular Mechanisms

Autophagy is an evolutionarily conserved degradation pathway in eukaryotic cells that relies on lysosomes and helps maintain cellular homeostasis and respond to various stresses [12,56]. Originally noted in the 1950s as double-membraned vesicles that engulf cytoplasmic material, autophagy gained recognition with the discovery of autophagy-related (ATG) genes in yeast in the early 1990s [56,57]. These discoveries demonstrated that autophagy extends beyond random self-degradation and instead exists as a highly regulated, multi-step system for the degradation of proteins with longevity, defective organelles, and complex molecular entities. Beyond its role in cell turnover, autophagy is strongly induced by nutrient starvation, hypoxia, infection, or chemotherapeutic insult, enabling the cell to recycle its internal resources for energy and biosynthetic needs [57,58].

In essence, the autophagy pathway unfolds in five interrelated stages: initiation, nucleation, elongation, closure, and lysosomal degradation, each of which is regulated by distinct ATG protein complexes [57,59]. This process starts with the activation of the ULK1 kinase complex, which responds to upstream signals from nutrient and energy status, mediated by mammalian target of rapamycin complex 1 (mTORC1) and AMP-activated protein kinase (AMPK) [60,61]. Following ULK1 activation, phagophore nucleation is mediated by the Beclin-1-VPS34 complex, resulting in the production of phosphatidylinositol-3-phosphate (PI3P), which recruits initial autophagic effectors to specific endoplasmic reticulum-associated structures, referred to as omegasomes, located in the cell [59,60]. Two ubiquitin-like protein conjugation systems facilitate membrane elongation and closure: the ATG12-ATG5-ATG16L1 complex and LC3 lipidation. The cleaved LC3-I is conjugated to phosphatidylethanolamine to form LC3-II, which is tightly bound to autophagosomal membranes and is considered a gold-standard marker of autophagic flux [62,63].

After closure of phagophores, late autophagosomes move on microtubules to lysosomes, and membrane fusion by SNARE proteins and Rab GTPases produces autolysosomes [64]. Lysosomal hydrolases break down the cargo protein inside into building block amino acids, lipids as fatty acids, and sugars, all of which are reused for anabolic activity and the generation of ATP [64]. In addition to removing dysfunctional components, autophagy maintains cellular metabolism under stress [13,58,65]. This study underscores the critical interplay between cargo sequestration and lysosomal degradation in both physiological and disease contexts [14,17].

The process of autophagic flux is regulated by various nutrient and stress sensors that control the mTORC1 and AMPK pathways. Under nutrient-rich conditions, mTORC1 activates ULK1 by phosphorylation, thereby inhibiting autophagy and stimulating anabolic growth [61,66]. Nutrient or growth factor deprivation leads to decreased mTORC1 activity, which, in turn, causes dephosphorylation and activation of ULK1. Simultaneously, the activation of AMPK under energy stress leads to ULK1 phosphorylation, further increasing autophagy [60,66]. Hypoxia activates HIF-1α, which regulates the expression of BNIP3 and BNIP3L, displacing Bcl-2 from Beclin-1 and initiating autophagy under hypoxic conditions [67]. Reactive oxygen species (ROS) produced by malfunctioning mitochondria can oxidize and suppress ATG4, thereby promoting LC3 lipidation and accelerating autophagosome formation [68].

Cells also employ selective forms of autophagy to eliminate specific substrates, such as mitochondria (mitophagy), peroxisomes (pexophagy), aggregated proteins (aggrephagy), lipid droplets, in addition to bulk degradation (lipophagy), and intracellular invading bacteria (xenophagy) [69]. Cargo receptors such as p62/SQSTM1, NBR1, and OPTN bind ubiquitinated cargo and interact with LC3. This ensures the selective engulfment of cargo [62]. PINK1 controls mitophagy by means of Parkin-mediated ubiquitination of injured mitochondria, therefore enlisting autophagy equipment to preserve mitochondrial quality control. This process resensitizes cells to chemotherapeutic drugs and compromises their capacity for tumor initiation by inhibiting selective autophagy in these subpopulations [23].

**Figure 2:**
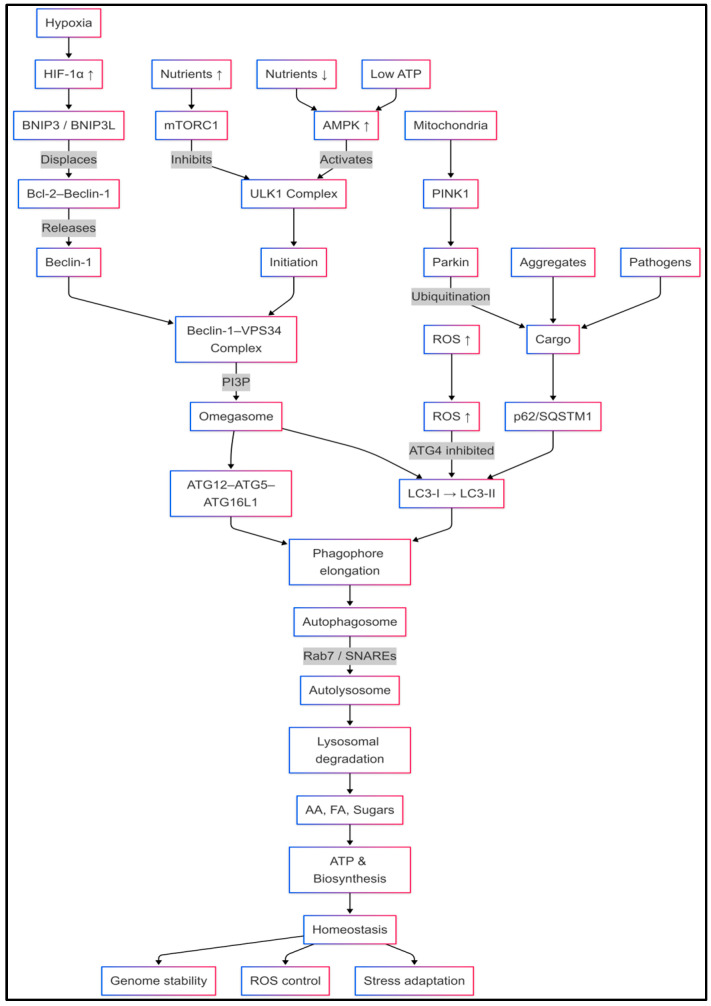
Molecular mechanisms regulating autophagy. Overview of the canonical autophagy pathway and regulation. In the presence of nutrients, the mechanistic target of rapamycin complex 1 (mTORC1) negatively regulates the Unc-51-like kinase 1 (ULK1) complex (ULK1/ATG13/FIP200/ATG101) to prevent autophagy initiation. However, upon nutrient starvation, AMP-activated kinase (AMPK) is activated, phosphorylating and stimulating the ULK1 complex to induce autophagy. Hypoxia triggers the release of Beclin-1 from Bcl-2, leading to the formation of class III phosphatidylinositol. The elongation of the phagophore involves the ATG12-ATG5-ATG16L1 complex and LC3 lipidation, from LC3-I to LC3-II. The mature autophagosomes fuse with lysosomes, thus forming the autolysosomes. It is at this site that cargo degradation and recycling take place. In addition, the process of selective autophagy is mediated by adaptor molecules, such as p62/SQSTM1. The upward (↑) and downward (↓) arrows show upregulation and downregulation, respectively.

Physiologically, autophagy is essential in embryogenesis, organogenesis, and the upkeep of stem cells. Mice deficient in Atg5 and Atg7 die during the perinatal stage with neurodegenerative and abnormal hematopoiesis. These findings underline the significance of autophagy in cell differentiation and tissue viability [70]. In immunology, autophagy promotes antigen presentation by dendritic cells (DCs); it controls the function of the inflammasome on macrophages and aids in clearing intracellular pathogens, thereby interacting with innate and adaptive defense systems [71]. A spectrum of human illnesses is caused by disrupted autophagy: insufficient autophagy leads to cellular dysfunction in neurodegenerative conditions, including Alzheimer’s disease and protein aggregation. Parkinson’s disease, in which, in metabolic syndrome, excessive or insufficient autophagy in hepatocytes worsens steatosis and insulin resistance [72] (Figure 2).

## 5. Autophagy in Cancer

Autophagy has dual roles in cancer. Early in tumorigenesis, autophagy functions as a tumor suppressor by keeping genome integrity, limiting oxidative stress, and preventing the buildup of damaged organelles [65,73]. Early in carcinogenesis, autophagy is initiated in response to cell protection. However, once tumors are established, autophagy supports survival under starvation stress and chemotoxicity and propels tumor growth, metastasis, and immunological evasion [65,74]. In TNBC, autophagy helps maintain cancer stem cells alive and induces increased PD-L1 expression and other immunosuppressive factor production in the TME, which helps to inhibit the immune response [75].

As with many biological processes implicated in both health and disease, autophagy is an interesting but also challenging target for drug development. The pharmacological agent, including chloroquine (CQ), and the genetic silencing of the ATG genes have shown promising preclinical effects in TNBC models with the induction of chemosensitivity and inhibition of tumor growth [17,76,77]. Although these agents have been evaluated clinically, the acidifying late-stage chloroquine and hydroxychloroquine lysosome inhibitors have downsides, including immunosuppression and off-target toxicity [74,78]. Compounds in development that target specific autophagy modulators, such as VPS34, ATG4B, or ULK1, have shown greater specificity, although the best combinations with chemotherapy or targeted therapies have yet to be determined. The equilibrium between autophagy’s roles in promoting cell death or survival is context-dependent. Achieving tumor-specific modulation calls for sophisticated approaches, such as synthetic lethality screens and nanoparticle delivery [76,79,80,81].

**Figure 3:**
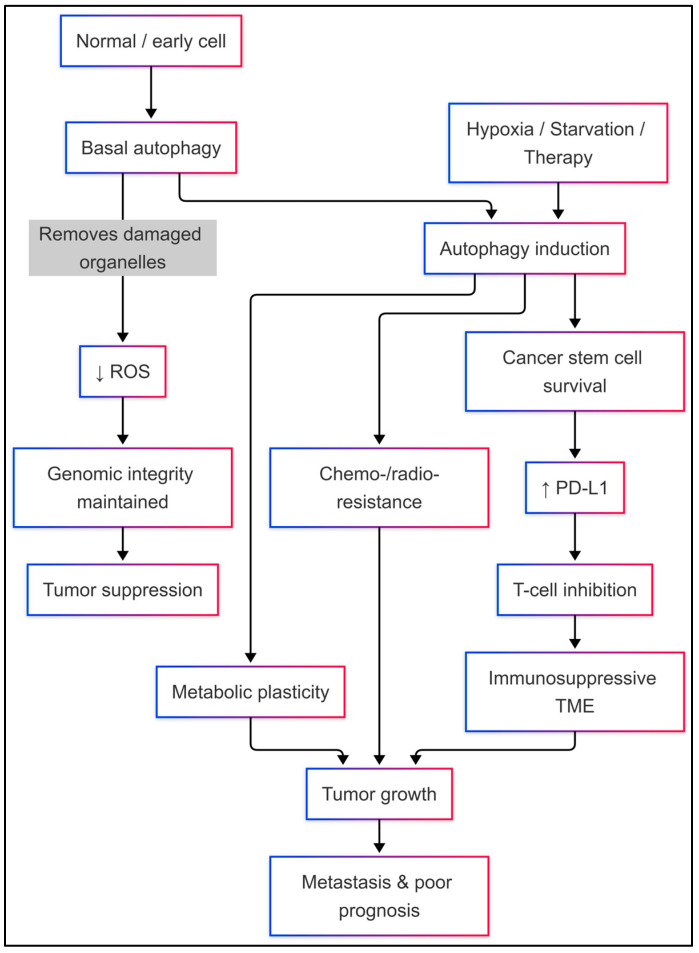
Dual role of autophagy in cancer progression. Context-dependent role of autophagy in tumorigenesis. In normal or early-stage cells, basal autophagy preserves proteostasis, limits oxidative stress, and maintains genomic stability, contributing to tumor suppression. In established tumors, stressors such as hypoxia, nutrient deprivation, and therapy induce sustained autophagy activation. In this context, autophagy supports metabolic adaptation, promotes survival, enhances therapy resistance, and contributes to cancer stem cell maintenance. In TNBC, elevated autophagy has been associated with increased PD-L1 expression and immunosuppressive TME, facilitating tumor progression and poor clinical outcomes. The upward (↑) and downward (↓) arrows show upregulation and downregulation, respectively.

Autophagy, in essence, is a fundamental, highly controlled process that balances cellular breakdown and renewal [56,58,65]. Its functions range from immunity and developmental homeostasis to tumor suppression and, ironically, tumor promotion under stress [65,74]. In addition, emerging evidence also shows that autophagy is involved in the process of chemoresistance, immune escape, and the survival of cancer stem cells, thereby establishing the importance of the autophagy axis in the process of therapy resistance and disease pathogenesis [17,75,76,77]. Next-generation translational research needs to fine-tune modulators of selective autophagy, capitalize on predictive biomarkers, and construct combination therapy that balances at an optimum level autophagy’s heterogeneity with the goal of deriving maximal clinical [74,76,77,82] (Figure 3).

## 6. Role of Autophagy in TNBC Chemoresistance

In chemoresistant phenotypes, TNBC cells maintain elevated autophagic flux as an essential survival mechanism. Exposure to anthracyclines, taxanes, or platinum compounds activates upstream stress sensors, thereby activating the AMPK–ULK1 signaling cascade, which relieves the mTORC1-mediated suppression, while BECLIN1-VPS34 complexes regulate the formation of vesicles involved in the lipidation of LC3 [29,83]. This recycling process maintains mitochondrial integrity through mitophagy, reduces ROS production, and maintains metabolic homeostasis by recycling amino acids and nucleotides [16,84]. An additional but less apparent function is the clearance of cytosolic double-stranded DNA released from damaged mitochondria and nuclei, which suppresses cyclic GMP-AMP synthase stimulator of interferon genes (cGAS-STING) pathway activation, attenuating type I interferon output and dampening anti-tumor immune surveillance [85].

Resistant TNBC cell cultures tend to naturally exhibit high levels of basal autophagy before any treatment, mediated by hypoxia-induced expression of BNIP3/BNIP3L and ROS signaling, and metabolic plasticity that can readily scale up autophagy levels in response to treatment pressure [16]. Such a pre-existing state can protect the bulk tumor cell population but also cancer stem-like cells, which exploit it to survive the initial onslaught of drugs and then repopulate the tumor [86,87]. Furthermore, there is a link between autophagy and epithelial–mesenchymal transition (EMT), which provides metabolic precursors for cytoskeleton remodeling and anoikis resistance, thus connecting drug persistence with metastatic competence [44,88].

**Figure 4:**
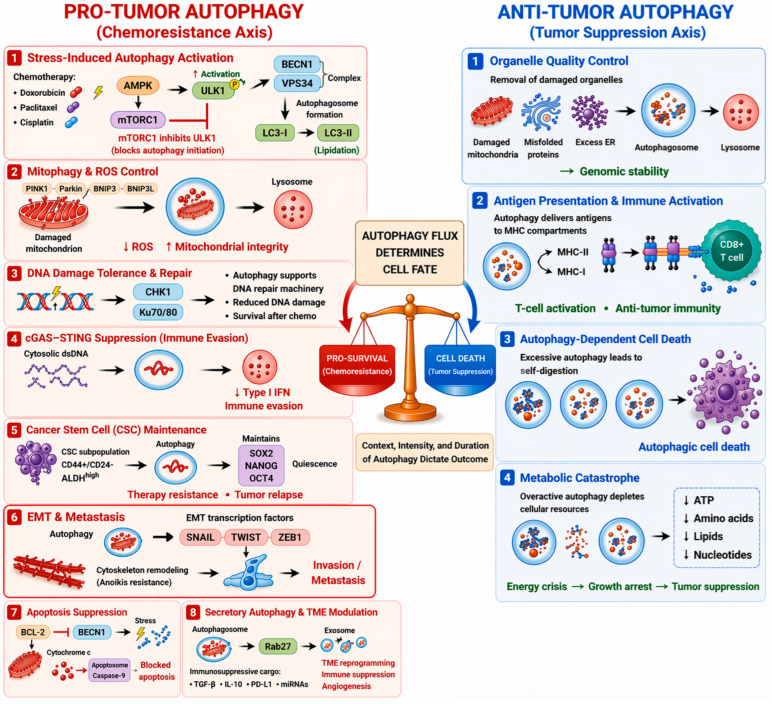
Autophagic role duality in TNBC: chemoresistance against anti-tumor activity. The proposed mechanism shows context-dependent regulation of autophagy in TNBC, with the dynamics of autophagic flux playing a significant role in determining cell fate. *Pro-tumorigenic autophagy (**left diagram**):* 1. Cellular stress due to chemotherapy induces the activation of AMPK–ULK1 signaling and BECN1-VPS34 complex to form an autophagosome through LC3 lipidation, while mTORC1 acts as a negative autophagy regulator. 2. Selective clearance of dysfunctional mitochondria by mitophagy takes place through PINK1/Parkin and BNIP3/BNIP3L mechanisms and helps in decreasing the ROS generation and maintaining mitochondrial function. 3. DNA damage repair is linked with CHK1 kinase and Ku70/80 protein complex. 4. Cytosolic DNA degradation results in inhibition of the cGAS-STING pathway and downregulation of type I interferon responses. 5. Autophagy sustains the cancer stem cell state, namely SOX2, NANOG, OCT4, and provides for cellular quiescence. 6. EMT process is enhanced by SNAIL, TWIST, and ZEB1, contributing to cytoskeletal remodeling and anoikis resistance. 7. Association of BCL-2 and BECN1 prevents apoptosis by blocking caspase activity. 8. Rab27-mediated secretory autophagy plays a role in immunosuppressive factor release. *Anti-tumorigenic autophagy (**right diagram**):* 1. Maintains organelle homeostasis that supports genome stability. 2. Increases antigen presentation via MHC-mediated pathways, thus activating CD8+ T cells. 3. Uncontrolled autophagy may lead to autophagic cell death. 4. Autophagy leads to metabolic depletion that results in an energy crisis and subsequent growth arrest. The upward (↑) and downward (↓) arrows show upregulation and downregulation, respectively. This schematic was created with the assistance of AI-based visualization tools.

The interaction of apoptotic regulators further enhances this protective response. The dissociation of BCL-2 and BECLIN-1 by stress-induced signaling pathways induces autophagy and increases the threshold for apoptosis, and secretory autophagy through the release of exosomes by Rab27 further changes the TME to have pro-survival and immunosuppressive properties [58,89,90]. In addition, autophagy plays a role in DNA repair by maintaining nucleotide pools and recruiting critical repair proteins, such as CHK1 and Ku70/Ku80, to facilitate recovery from chemotherapeutic-induced damage [84]. These combined functions of autophagy make this process an efficient and multifaceted survival network (Figure 4), and accurate biomarker-mediated inhibition of this process has the potential to disrupt this network and re-establish therapeutic sensitivity [26,29].

### 6.1. Cancer Stem Cells and Autophagic Quiescence

A persistent obstacle in TNBC treatment is the ability of a small, highly adaptable subpopulation of CSCs to withstand chemotherapy and later regenerate the tumor. These cells, typically characterized by CD44/CD24 profiles or elevated aldehyde dehydrogenase (ALDH) activity, can enter a metabolically subdued, non-proliferative state known as quiescence during chemotherapeutic stress [86,87]. Quiescence shields CSCs from the killing mechanisms of most cytotoxic agents, which preferentially target rapidly dividing cells. Autophagy is a central enabler of this state, functioning as both a metabolic life-support system and a quality-control mechanism [86].

Quiescent CSCs rely on autophagy to maintain their long-term survival, especially in the nutrient-deprived and hypoxic environments typical of tumor niches [23,91]. Mitophagy, a selective form of autophagy, which includes a type of autophagy, has been found to perform an important function in maintaining cellular homeostasis [75]. This group includes PINK1, Parkin, and BNIP3/BNIP3L, all of which are involved in mitophagy. This process helps degrade mitochondria, thereby avoiding the production of excessive ROS, which may trigger senescence and/or apoptosis [75]. In addition, the degradation of oxidatively damaged proteins through autophagosome-lysosome degradation helps to maintain proteome integrity [92]. Recycling of amino acids and lipids, therefore, plays an important role in providing substrates for vital biosynthetic and energy-yielding metabolic pathways, thus helping to maintain the expression of stemness-associated transcription factors such as SOX2, NANOG, and OCT4, which are vital for CSC renewal [75,92].

Evidence of this dependency comes from functional studies demonstrating that genetic suppression of autophagy genes such as ATG5, ATG7, and BECN1 significantly impairs tumor sphere formation, ALDH activity, and in vivo tumor initiation in TNBC models [23,91]. On the other hand, pharmacologic inhibition of autophagy using chloroquine analogs or VPS34 inhibitors will reduce autophagic flux, thereby activating and sensitizing cancer stem cells (CSCs) to chemotherapy-induced apoptosis [92]. However, autophagy’s role in CSC fate is context dependent. In some TNBC contexts, sustained high flux preserves quiescence and stemness; in others, excessive autophagic activity can drive differentiation or even trigger autophagy-dependent cell death. This dual nature demands biomarker-guided therapeutic strategies that monitor both ALDH activity and LC3/p62 turnover to determine whether flux supports survival or pushes cells toward vulnerability [87].

TME also plays an active role in maintaining the autophagic quiescence of CSCs. Hypoxic niches induce HIF-1α, which upregulates BNIP3/BNIP3L, thus linking hypoxia with the induction of mitophagy in CSCs [24,93]. Moreover, cytokines like TGF-β and IL-6, secreted by the stroma, also induce the EMT-like processes and autophagy, leading to the formation of CSCs [44]. It has also been found that in preclinical research, the inhibition of these external signals by using a combination of TGF-β inhibitors and autophagy inhibitors disrupted the quiescence of CSCs and reduced the amount of minimal residual disease and metastatic colonies that developed after chemotherapy [24,92,93].

Collectively, CSC retention in TNBC is mediated by a close interplay between quiescence and autophagy, where Zhao et al. (2025), Vazquez-Martin et al. (2010) and Jia et al. (2025) have shown that this interplay is crucial in allowing CSCs to survive metabolic and genotoxic challenges, evade the host immune system, and act as a reservoir of latent relapse [75,87,92]. Breaking this cycle will likely require precisely timed autophagy inhibition, coordinated with chemotherapy pulses or differentiation therapies, to flush CSCs out of dormancy and into a state of therapeutic vulnerability [75,91].

### 6.2. Genetic Knockdown and Pharmacological Inhibition of Autophagy

A substantial body of evidence supports the causal role of autophagy in TNBC chemoresistance, as demonstrated by genetic inhibition of the autophagy cascade and pharmacological intervention at crucial nodes. The genetic inhibition of vital autophagy gene products, i.e., ULK1, BECN1, ATG5, and ATG7, in the process of TNBC chemoresistance, leads to the reduced production of autophagosomes, LC3-II, and P62/SQSTM1 protein, thus reaching its inhibition [41,45]. These genetic alterations render cells re-sensitized to a variety of chemotherapeutic agents, including doxorubicin, paclitaxel, and cisplatin, through increased mitochondrial outer membrane permeabilization, release of cytochrome C, and activation of caspase 3/7 [45]. Importantly, mechanistic validation is supported by upstream pathway target research, such as NSD2 histone methyltransferase inhibition, which directly stimulates ULK1 transcription via the histone modification H3K36me2 and suppresses autophagic flux, tumor growth, and metastatic potential, both in vitro and in vivo. This study shows that, in NSD2-high TNBC, autophagy is not just a downstream survival mechanism but is transcriptionally dependent [41].

Pharmacologic autophagy inhibition offers complementary insights. Early stage inhibitors target the initiation complex, ULK1 inhibitors (SBI 0206965) or VPS34 inhibitors (SAR405, PIK III), blocking phagophore formation and autophagosome nucleation [45]. The late-stage inhibitors chloroquine and hydroxychloroquine target lysosomes to increase pH, thereby inhibiting fusion between autophagosomes and lysosomes and promoting degradation [74]. In TNBC models, these compounds, in combination with chemotherapy, prolong DNA double-strand breaks, disrupt mitochondrial recovery, and lower apoptotic thresholds [45]. The stage of the inhibitor may affect the immunological outcomes, as the VPS34 inhibitor increases the expression of chemokines (CXCL10) through the NF κB pathway, a factor that may improve the recruitment of T cells, while the late-stage inhibitor may affect the endolysosomal trafficking, thereby improving or reducing the immunological outcomes [74].

Targeting metabolic genes also provides experimental evidence for the role of these genes in the development of the disease. The inhibition of the SLC25A17 gene, a transporter on the peroxisome, increases oxidative stress, which induces autophagy and apoptosis while inhibiting EMT and metastasis [51]. Interestingly, pharmacologic inhibition of autophagy with 3-methyladenine (3-MA) rescues cells from SLC25A17 knockdown-induced apoptosis, indicating that, in this context, autophagy contributes to cell death rather than survival. This underscores the point that autophagy’s functional role can be dichotomous, and therapeutic manipulation should be guided by biomarkers of flux directionality [94].

There is also growing interest in leveraging autophagy inhibition to potentiate immunotherapy and radiotherapy. In certain TNBC models, suppressing autophagy increases cytosolic DNA levels, amplifying cGAS-STING and NF-κB pathways to promote a pro-inflammatory TME [45]. Nevertheless, these effects are not uniform across all molecular subtypes, as inhibition of autophagy induces PD-L1 upregulation, indicating that rational use of a triple combination, such as genotoxin + autophagy inhibitor + PD-1/PD-L1 blockade, will be required and necessary depending on the subtype based on its genome and immunologic subtype [95].

In conclusion, whereas genetics and pharmacology have definitively established that in TNBC, autophagy is not “passive” but “active”, influencing cell survival [41,45,74]. This process, by targeting multiple aspects of the autophagic pathway, with measurements of baseline flux, dsDNA content, and immune activation, will reveal tumor-specific vulnerabilities, as demonstrated by Zhou et al. (2024) and Zhou & Zhou (2025) [51,95]. The key will be to determine whether autophagy functions as a ‘shield’ or ‘sword’ for the tumor [45,74].

### 6.3. Autophagy Interaction with Apoptosis, EMT, and DNA Damage Repair

Autophagy’s contribution to TNBC chemoresistance is amplified by its extensive crosstalk with apoptosis, EMT, and DNA damage repair (DDR) networks. In the context of apoptosis, autophagy generally serves as a cytoprotective mechanism. When cells are exposed to chemotherapy-induced stress, autophagy removes damaged mitochondria before they can release cytochrome c, effectively blocking activation of the intrinsic apoptotic pathway [58,84]. The anti-apoptotic BCL2 and BCLXL proteins interact with BECLIN1 and inhibit autophagy in basal conditions, whereas the activation of stress kinases such as JNK, which phosphorylates BCL2, releases BECLIN1 and activates autophagy [58]. This mode of autophagy activation is referred to as the “cytoprotective” effect, which, in turn, raises the threshold of apoptosis, allowing the tumor cells to survive in the face of lethal genotoxic and oxidative stress. However, in extreme cases, this balance can be reversed, where the activation of caspases leads to the cleavage of BECLIN1 or ATG, resulting in apoptosis, or when autophagy is overactivated, resulting in autophagic cell death. In chemoresistant TNBC, however, the balance is usually tipped toward pro-survival autophagy, delaying or preventing apoptosis during treatment [84].

In parallel, autophagy plays a role in the acquisition and maintenance of EMT properties. EMT equips TNBC cells with increased motility, invasiveness, and resistance to anoikis, thus favoring the dissemination of metastases. Stress signals, including TGF-β, nutrient starvation, and hypoxia, co-induce EMT transcription factors (SNAIL, TWIST, and ZEB1) and autophagy regulators. This connects how metabolism changes with the ability of an organism to alter its physical traits [44,88]. The activation of ULK1 through epigenetic changes caused by NSD2 is connected to a rise in autophagic flux and a simultaneous increase in mesenchymal markers like N-cadherin in TNBC [41]. Autophagy, by maintaining metabolic and redox balance, enables EMT-programmed cells to survive detachment and traverse challenging microenvironments. Conversely, in certain contexts, autophagy can degrade EMT-promoting proteins (such as SNAIL) and restrain metastasis, illustrating that the outcome of autophagy-EMT interplay is context-dependent but, in chemoresistant TNBC, predominantly pro-migratory [88].

Autophagy also plays a significant role in DDR, which directly impacts the efficacy of chemotherapeutic agents. Efficient repair of drug-induced DNA double-strand breaks and other lesions allows TNBC cells to survive genotoxic therapy. Autophagy maintains ATP and nucleotide levels in cells while it removes damaged repair proteins, and it controls how important repair proteins, such as XPC for nucleotide excision repair and Ku70/Ku80 for non-homologous end joining repair to the process of DNA repair [84,96]. Active autophagy flux may also facilitate homologous recombination by enhancing CHK1 activation and maintaining replication fork stability under genotoxic stress. The efficient repair of DNA damage by autophagy reduces the cytotoxic effects of chemotherapy and allows for survival [96].

Beyond its role in repair, autophagy influences the immunogenic consequences of DNA damage. Normally, unrepaired nuclear or mitochondrial DNA fragments can leak into the cytosol and activate the cGAS-STING pathway, driving type I interferon production and antitumor immune recruitment. Elevated autophagic activity degrades this cytosolic DNA, thereby blunting cGAS-STING signaling and reducing immune-mediated clearance [85]. In certain situations, blocking autophagy restores immune activation, thereby increasing PD-L1 expression through immune checkpoint regulation. This discovery supports the development of triplet therapies that combine chemotherapy with autophagy inhibitors and PD-1/PD-L1 blockade [95].

The interconnection of autophagy with three distinct processes, which include apoptosis suppression and EMT-driven plasticity, and DDR efficiency, results in a resistance system that functions as a protection mechanism for TNBC [41,58,84,88,96]. This has enabled the tumor cell not only to survive acute cytotoxic stress but also to become metastatic while evading the host’s immune system [85,95]. Simultaneous interruption of these autophagy-related cell-survival mechanisms has been shown to be effective in managing chemoresistance (Figure 4) [41,95].

## 7. Autophagy and Immune Resistance in TNBC

**Figure 5:**
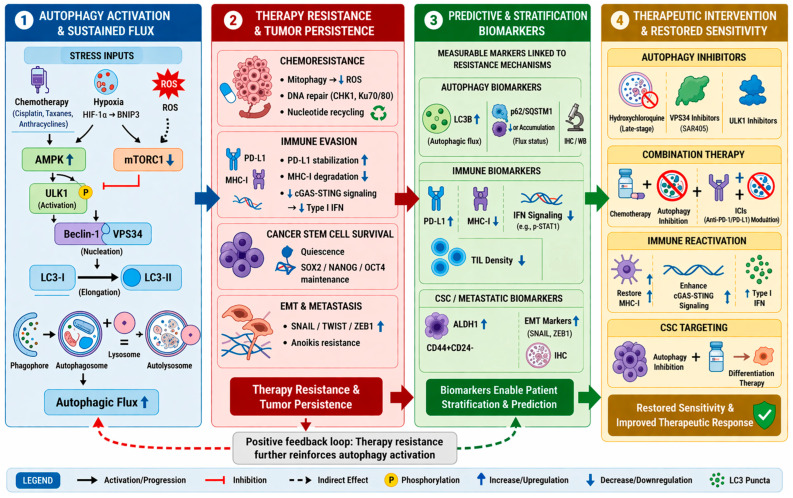
Autophagy-dependent pathways connecting stress adaptation, treatment resistance, biomarker stratification, and targeted treatment approaches in TNBC. The current figure describes how autophagy influences TNBC, starting from activation up to targeting. (1) *Autophagy activation and flux:* Cancer treatments, hypoxia-induced stress (HIF-1α-BNIP3 pathway), and production of ROS stimulate autophagy. Stress due to energy depletion activates AMPK and inhibits mTORC1, leading to derepression of ULK1 and the initiation of autophagy via the Beclin-1-VPS34 pathway. LC3-I to LC3-II conversion leads to the formation of autophagosomes, and lysosomal fusion is required to maintain autophagic flux. (2) *Therapy resistance and tumor persistence:* Autophagy increases cell survival by decreasing ROS through mitophagy, repairing DNA through CHIK1, and recycling metabolites. Immune escape is achieved through downregulation of PD-L1, MHC-I, and the cGAS-STING/type I interferon pathway. Cancer stemness properties are sustained by autophagy through SOX2, NANOG, and OCT4 proteins, while the EMT process is mediated by SNAIL, TWIST, and ZEB1 proteins. (3) *Predictive biomarkers:* Important biomarkers include autophagy biomarkers like LC3B and p62/SQSTM1, as well as immune biomarkers including PD-L1, MHC-I, IFN signaling, and TILs. Other important factors related to stemness/metastasis, as well as EMT biomarkers, are also relevant for patient stratification. (4) *Therapeutic intervention:* The most promising treatments involve the use of drugs that inhibit autophagy (e.g., hydroxychloroquine, VPS34 inhibitors, and ULK1 inhibitors). Such a strategy can effectively induce sensitivity to immunotherapy and chemotherapy by improving antigen processing, activating the cGAS-STING pathway, increasing IFN production, and inhibiting CSCs. A positive feedback loop indicates that therapy resistance reinforces autophagy activation. This schematic was created with the assistance of AI-based visualization tools.

The various biological subtypes of TNBC, including basal-like, mesenchymal, and immunomodulatory, lead to aggressive clinical progression and complicate treatment [11]. Despite its relatively higher tumor mutational burden and immunogenicity compared to hormone receptor-positive disease, TNBC remains challenging to treat, with ICIs producing durable benefit in only a subset of patients, many of whom develop resistance over time [49]. This paradox underscores the presence of potent immune resistance mechanisms within the TNBC TME. Among these mechanisms, autophagy plays a distinct role. This highly conserved form of lysosomal degradation, as it is, has emerged as an important aggressive tumor behavior in tumor-immunity dynamics. In order to resolve the apparent paradoxical nature of autophagy’s role in TNBC due to its different contexts, it is necessary to have a unified model based on three important perspectives: (i) basal autophagy vs. treatment-induced autophagy, (ii) autophagy in tumor cells vs. autophagy in immune/stromal cells, and (iii) protective vs. destructive biological consequences of autophagy. It modulates key components of antigen presentation, cytokine signaling, the extracellular matrix milieu, and even immune cell function (Figure 5) [52,95]. Resistance to the immune system in TNBC arises from both the tumor’s intrinsic properties and dysfunctional immune cells. Intrinsic properties involve autophagy and the destruction of immune signals, while resistance associated with immune cells comprises faulty antigen presentation, dysregulation of dendritic cells, and T cell exhaustion (Table 1). Distinguishing these layers is essential for interpreting the role of autophagy in immune escape.

At the tumor cell-intrinsic level, autophagy can suppress anti-tumor immunity by degrading immunogenic cargo such as damage-associated molecular patterns (DAMPs), including HMGB1 and ATP, which are critical for DC activation and T cell priming [28,101]. Basal autophagy limits the accumulation of cytosolic DNA and, consequently, attenuates cGAS-STING-mediated type I interferon signaling (Figure 5), which plays an important role in immune surveillance [10]. In this context, basal autophagy primarily supports tumor cell homeostasis and immune evasion, whereas therapy-induced autophagy triggered by chemotherapy, radiotherapy, or immunotherapy may either enhance immunogenic cell death or promote adaptive resistance depending on treatment timing and intensity. In TNBC, this suppression alters PD-L1 expression and reduces the presence of tumor-infiltrating lymphocytes, which leads to better immune defense against tumors [102]. Conversely, in certain therapeutic contexts, such as chemotherapy-induced immunogenic cell death (ICD), autophagy is required for optimal ATP release and antigen cross-presentation, illustrating its context-dependent role [28]. Functionally, these opposing effects reflect the dual nature of autophagy, where moderate activation is often cytoprotective and supports tumor survival, whereas excessive or dysregulated autophagy can contribute to cytotoxic outcomes and enhance anti-tumor immunity. One of the most compelling mechanistic links between autophagy and immune resistance in TNBC involves the ECM glycoprotein Tenascin-C (TNC). Under normal conditions, TNC is selectively degraded via p62/SQSTM1-mediated autophagy following Skp2-catalyzed Lys63-linked ubiquitination at Lys942 and Lys1882 [52]. When autophagy is impaired, TNC accumulates in the ECM, creating a physical and biochemical barrier that excludes cytotoxic CD8+ T cells and dampens their effector function. Clinically, high TNC expression is associated with low LC3B levels, reduced TIL density, and a poor prognosis in TNBC patients. Restoring autophagic degradation of TNC or directly targeting TNC can re-sensitize tumors to T cell-mediated killing and enhance responses to PD-1/PD-L1 blockade [52].

Importantly, autophagy operates across multiple cellular compartments within the TME. While tumor cell-intrinsic autophagy often facilitates immune evasion, autophagy in immune and stromal cells can either support or suppress anti-tumor immunity depending on the specific cell type and microenvironmental cues. Autophagy plays a role in regulating the function of immune cells in TME (Figure 5). For DCs, autophagy plays a role in antigen processing and cross-presentation, especially under conditions of metabolic and oxidative stress [103]. The dysregulation of autophagy in DCs by tumor-derived factors may compromise their ability to induce CD8+ T cells, leading to primary resistance against ICI [95]. In macrophages, autophagy plays a role in polarization states, with autophagy in lactate-rich, hypoxic niches of TNBC cells affecting HIF-1α and mTOR signaling pathways, leading to the maintenance of M2-like macrophages, which secrete immunosuppressive cytokines and are characterized by high [50,104]. In T cells themselves, autophagy maintains mitochondrial quality control during chronic antigen exposure, but excessive activation can promote exhaustion by limiting anabolic signaling and IFN responsiveness [105].

The metabolic background of TNBC is related to immune resistance through autophagy. TNBC has a glycolytic phenotype, which means that there is a high production of lactate, leading to a low pH in the TME, which compromises the activity of immune effector cells [106]. In addition, lactate is responsible for the lactylation of histones and proteins, which modulates the transcription of genes in cancer and immune cells, leading to immune evasion, angiogenesis, and metastasis [104]. Autophagy is related to the metabolic phenotype of TNBC, as it is responsible for providing substrates to maintain the glycolytic phenotype under hypoxic conditions. Inhibiting lactate metabolism via LDH inhibition or lactate oxidase, as well as modulating autophagy, has demonstrated synergistic effects with PD-L1 inhibition in preclinical models of TNBC [50,104].

Regulation of gene expression by noncoding RNAs (ncRNAs) is critical for controlling immune resistance and autophagy. In particular, the long noncoding RNA, RMST, serves as a competitive endogenous RNA (ceRNA) that ‘sponges’ a microRNA (miRNA), in this case miR-4295, leading to the ITPR1 derepression. Augmentation of ITPR1 expression increases Ca^2+^ equilibrium between the endoplasmic reticulum and mitochondria, thereby promoting autophagy. In TNBC cells, it has been demonstrated that this control pathway inhibits cell proliferation and migration, while at the same time inducing apoptosis and autophagy. Although autophagy amplification has been investigated in tumor cell biology, it could indirectly affect antigen presentation and ICD [104].

From a therapeutic point of view, these findings indicate that modulation of autophagy in TNBC needs to be context-dependent and time-controlled. Tumor cell-specific inhibition of autophagy during antigen-releasing treatments may augment ICD and antigenicity, while preserving or even potentiating autophagy in DCs and T cells to retain immunocompetence [28,95]. Targeting immune barriers controlled by autophagy is important, with TNC representing one such barrier; moreover, modulating macrophage autophagy to favor M1 polarization offers a potential strategy to overcome this immunosuppressive obstacle. In addition, the combination of autophagy modifications with metabolic therapies has the potential to decrease lactate levels. Moreover, it is crucial to use a combination of biomarker-based strategies, including LC3B/p62, TNC, TIL density, IFN gene signatures, and lactate/lactylation for patient stratification in upcoming clinical trials [52,99].

Taken together, these observations highlight that the role of autophagy in TNBC is not inherently pro-survival or anti-tumor, but is dictated by the interplay between autophagy type, cellular compartment, and therapeutic context. Therefore, in the context of TNBC, it is imperative to say that the role of autophagy as a master regulator of immune resistance in the disease involves tumor-intrinsic, immune-cell-intrinsic, and metabolic mechanisms. The dualistic role of autophagy in cancer requires precise targeting to convert it from a tumor shield into a facilitator of anti-tumor immunity. Rationally designed, biomarker-guided strategies that integrate autophagy modulation with ICIs and metabolic reprogramming hold promise for overcoming immune resistance and improving outcomes in TNBC [7,47].

### 7.1. Autophagic Control of PD-L1 Expression and Trafficking

PD-L1 is a transmembrane immune checkpoint protein that binds to PD-1 on activated T cells, delivering inhibitory signals that suppress cytotoxic activity and promote immune tolerance within the TME [48]. In the context of TNBC, PD-L1 is reported to be overexpressed to a greater extent than in other subtypes of breast cancer. Specifically, it has been reported that 20% of TNBCs exhibit high PD-L1 positivity [48,102]. This overexpression is significant because PD-L1 levels influence the prognosis and treatment with ICIs such as atezolizumab and pembrolizumab [47]. PD-L1 is dynamically and tightly regulated at the transcriptional, post-transcriptional, and post-translational levels. Autophagy has been reported to be a significant post-translational regulator of PD-L1 expression [54].

#### 7.1.1. Autophagy as a Regulator of PD-L1 Homeostasis

Cells rely on autophagy as a degradative pathway in which lysosomes dismantle faulty organelles, misfolded proteins, and other unwanted components to sustain internal stability [107]. In cancer, this process is not limited to the usual “housekeeping” functions. Indeed, there is evidence that this process also has the capability of rerouting certain plasma membrane proteins, including key immune checkpoint molecules, into the lysosomal compartment for degradation. Several studies have shown that PD-L1 undergoes endocytosis and is then sorted into either recycling endosomes or autophagosome-lysosome compartments, where it can be degraded. The balance between these pathways determines the steady state level of PD-L1 on the tumor cell surface [100,108].

PD-L1 appears to be affected differently by autophagy in TNBC. Elevation of autophagic flux reduces surface expression of PD-L1, thereby rendering tumor cells prone to T cell-mediated death [100]. On the other hand, autophagy inhibition, whether pharmacologic (such as chloroquine and bafilomycin A1) or genetic (such as ATG5/ATG7 knockdown), has been shown in other tumor models to increase PD-L1 expression, often through ERK/JNK/c-Jun signaling or through stabilization of PD-L1. This implies that autophagy may play a role as a suppressor of PD-L1 expression [54].

#### 7.1.2. Molecular Mediators Linking Autophagy and PD-L1 Trafficking

PD-L1 does not move randomly within the cell; adaptor proteins and various post-translational changes carefully coordinate its trafficking from the membrane to lysosomes. Two proteins, CMTM6 and HIP1R, serve as key regulators that prevent the degradation of PD-L1 by the lysosome [109]. CMTM6 assists with recycling the receptor back to the cell membrane from the endosome, while HIP1R is responsible for the uptake of the receptor by the cell through the clathrin-mediated endocytic pathway and directs it to the lysosome. Autophagy plays a role by regulating the fate of the endocytosed PD-L1 receptor, either recycling it or degrading it [108,109].

In TNBC, loss of tumor suppressors such as PTEN can upregulate PD-L1 transcription via PI3K–AKT-mTOR signaling, but mTOR is also a master regulator of autophagy [48]. Hyperactivation of mTOR suppresses autophagy, potentially reducing PD-L1 degradation and increasing its surface stability. Conversely, mTOR inhibition can induce autophagy and promote PD-L1 turnover, although the net effect on immune evasion depends on the balance between PD-L1 degradation and other autophagy-mediated pro-survival effects [42].

#### 7.1.3. Crosstalk Between Autophagy and PD-L1 in the Immune Microenvironment

The impact of autophagy on PD-L1 is not limited to tumor cells. In antigen-presenting cells (APCs) such as dendritic cells and macrophages, autophagy can modulate PD-L1 expression in response to inflammatory cues [103]. IFN-γ stimulation induces PD-L1 transcription via JAK-STAT signaling, but autophagy can fine-tune the duration and magnitude of PD-L1 surface expression by controlling its degradation. In tumor-associated macrophages (TAMs), lactate-rich conditions common in TNBC due to the Warburg effect can suppress autophagy and stabilize PD-L1, reinforcing an immunosuppressive phenotype [50,104].

Understanding the autophagic control of PD-L1 opens new therapeutic avenues. Combining autophagy inducers with ICIs could show a potential reduction in PD-L1 surface levels and enhance T cell activity. However, because autophagy also supports tumor cell survival under stress, such strategies must be carefully timed and possibly restricted to “autophagy pulses” during ICI treatment [28]. As an alternative, selective inhibition of PD-L1 trafficking regulators like CMTM6 or HIP1R could be used to favor the autophagy-mediated degradation without broadly activating autophagy in tumor cells [108].

In TNBC, where PD-L1 expression is both a biomarker and a therapeutic target, the modulation of autophagy could help overcome the challenges of resistance, either acquired or primary. The use of the “biomarker-driven” strategy, including the measurement of the rate of autophagy flux, PD-L1 turnover, and regulators of trafficking, will be critical for the design of clinical trials [108].

### 7.2. Impact of Autophagy on Antigen Presentation and T-Cell Priming

Antigen presentation and priming of T lymphocytes are key events in orchestrating antitumor immunity; however, their disruption is one of the hallmark events of immune resistance in TNBC. Although TNBC tumors exhibit high tumor mutational burden and lymphocyte infiltration, the effectiveness of ICIs may vary across TNBC subtypes. This variability may result from defects in antigen presentation and the integrity of the priming cascade [7,47]. Both macroautophagic and non-canonical LC3-associated phagocytosis (LAP) play central roles in orchestrating antitumor immunity. Through their influence on antigen processing in tumor cells, MHC molecule transport, dendritic cell cross-presentation, and the inflammatory milieu, these pathways help shape the activation of CD8^+^ T cells [103,110].

Tumor-intrinsic autophagy has significant effects on antigenicity. It provides peptides for MHC class I presentation, yet it also takes away from proteasomal presentation, limiting the repertoire of peptides displayed [111]. Moreover, autophagy has been shown to degrade MHC class I molecules, as seen in pancreatic cancer, where NBR1-mediated autophagy reduced MHC class I expression, leading to reduced recognition by cytotoxic T cells, which was restored by inhibition of autophagy, thereby resensitizing tumors to immune attack [10]. These findings, although derived from non-TNBC settings, highlight a broader principle where high autophagy flux enables tumors to shield themselves from immune detection. Importantly, transient suppression of tumor autophagy during chemotherapy or radiotherapy enhances ICD by promoting ATP release and antigen availability, boosting the material available for cross-presentation [28,101].

Conventional type 1 dendritic cells (cDC1s), which depend on Batf3, play a critical role in activating CD8^+^ T cells. When these cells are dysfunctional, patients often exhibit poor responses to immune checkpoint blockade [112,113]. There are different ways in which autophagy supports DC biology, including the presentation of MHC-II for CD4^+^ T helper cells, regulation of vesicle trafficking to preserve peptides, and regulation of phagosomal maturation to balance preservation and destruction [103,114]. By contrast, LAP accelerates cargo degradation, limiting antigen persistence for cross-presentation and potentially skewing myeloid responses toward tolerance. This dichotomy underscores the need for cell type-specific modulation restraining LAP in tumor-associated phagocytes while maintaining macroautophagy in DCs to sustain their metabolic and antigen-processing capacity [115,116].

T-cell priming in TNBC is further limited by both the extracellular matrix (ECM) and the metabolic landscape of the tumor. A deficiency in autophagy stabilizes tenascin-C, creating a structural barrier against T-cell entry and the formation of antigen-presenting niches [100]. On the other hand, the WNT/β-catenin pathway in tumor cells suppresses the entry of cDC1, thus aborting cross-priming, while autophagy modulates these events via its regulation of cGAS-STING signaling and dendritic cell recruitment [53,113]. At the same time, glycolysis in tumor cells produces high levels of lactate, impairing the functionality of dendritic cells and driving macrophages toward an immunosuppressive state [50]. Tumor autophagy contributes to maintaining this glycolytic environment, suggesting that targeting autophagy might be beneficial in the reduction in lactate levels and the improvement of the antigen-presenting capacity. These metabolic and spatial barriers emphasize the point that the defects in the priming of T cells in TNBC are not only molecular but also structural, as related to the TME [104].

Taken together, these insights support a translational framework in which the modulation of autophagy is integrated into the therapeutic regimens used in immunotherapy. While the inhibition of tumor cell autophagy using short pulses during ICD-inducing therapies could augment the release of antigens and DAMPs, the inhibition of LAP activity specifically in myeloid cells could prolong the persistence of antigens, the maintenance of autophagy in DCs could sustain the functional fitness of these cells, and the use of metabolic modulators could counter the suppression caused by lactate. However, the indiscriminate inhibition of autophagy could lead to the suppression of the energy metabolism of DCs, while the activation of autophagy in tumor cells could result in the premature loss of tumor antigens. Therefore, it is likely that the therapeutic regimens that modulate autophagy with the most significant clinical potential are those that are able to sculpt autophagy differently in tumor and immune cells [111].

Operationalizing this approach could involve the use of composite biomarkers that include the measurement of autophagy flux (LC3B and p62/SQSTM1), MHC-I, DAMPs, cDC1, and spatial relationships between DC and T cells. This stratification could be used to classify TNBC tumors on the basis of their ability to support priming of T cells versus impaired priming [7]. Functional readouts such as ex vivo cross-presentation assays, T-cell receptor (TCR) clonality expansion, and interferon-stimulated gene induction will be key to assessing pharmacodynamic effects of autophagy modulation in clinical trials. Ultimately, the therapeutic goal is to choreograph autophagy states across tumor and immune compartments to maximize antigen generation and preservation, as well as T cell priming. In TNBC, in which immune resistance is often mediated by defective antigen presentation, such a targeted approach may be the solution to unleashing the true potential of ICIs [28,110].

### 7.3. Autophagy-Mediated Crosstalk with Myeloid Cells and the Cytokine Milieu

TNBCs acquire a microenvironment that is immunosuppressive in nature, which is mainly mediated by TAMs, MDSCs, dendritic cells, and neutrophils. The myeloid cells mediate the cytokine and chemokine networks, which in turn control the infiltration of immune cells and their killing activity in the tumor. Tumor cell autophagy plays the role of a key regulator of these interactions. It also regulates interactions with myeloid cells in the TME. Tumor autophagy regulates the release of DAMPs such as HMGB1 and IL-1β through secretory autophagy. These signals activate TLR4/RAGE receptors on myeloid cells and NF-κB transcriptional regulation of myeloid cell recruitment and polarization [117,118,119]. These signals exert context-dependent effects on immune activation in one situation, while they lead to suppressed immune activities through their impact on microenvironments in another case, which illustrates how autophagy regulates immune system activities through its complex mechanisms [120].

Autophagy is also involved in the regulation of innate sensing pathways, and the cGAS-STING pathway, which regulates the production of type I interferon (IFN-I), is particularly subject to this regulation. In TNBC, autophagy in tumor cells limits the accumulation of cytosolic DNA and enhances the degradation of STING through lysosomes, resulting in the suppression of IFN-I signaling [121]. Suppression of autophagy increases IFN-I and chemokine secretion, resulting in an increased accumulation of antigen-presenting myeloid cells and priming of T cells. Thus, autophagy helps to function as a “gain control” for immune activation in the TME, regulating the equilibrium between immune suppression and activation [45].

In myeloid subsets, autophagy acts as a cell-intrinsic orchestrator for its immune phenotype and function. TAMs have also been described to execute LAP to degrade phagocytic cargo efficiently while restricting inflammatory signaling, while maintaining an M2-like immunosuppressive phenotype with secretion of IL-10 and TGF-β [116]. The pharmacological inhibition of autophagy and/or LAP in TAMs has also shown the ability of TAMs to be repolarized towards a pro-inflammatory phenotype of M1 macrophages with increased IL-12 and TNF-α, along with decreased IL-10 and Arg1 [5]. Similarly, MDSCs also require autophagy to maintain viability and suppressive function under stressful TME. Tumor-derived HMGB1 reinforces this suppressive program by inducing autophagy via TLR4/RAGE signaling [122,123].

Another possible way by which immunosuppression [51,124,125]. DCs require autophagy for antigen processing and cross-presentation, but DC LAP has also been shown to limit cross-presentation by identifying antigens taken up from the tumor as ‘immunologically silent.’ Thus, while tumor autophagy has been shown to enhance the antigen pool through DAMPs and chemokines, myeloid-intrinsic autophagy or LAP has the potential to limit cross-priming [116,126].

Autophagy is known to have a critical role in the regulation of the cytokine cascade, which is an important factor in immune evasion. In TNBC, the IL-6 signaling pathway, which activates Stat3, is known to be associated with the regulation of CSCs, chemoresistance, and myeloid cell infiltration. Autophagy in tumors is known to have a role in the regulation of IL-6 secretion and signaling. This process creates an environment that protects against immune attacks by increasing M2 and myeloid-derived suppressor cells (MDSC) presence. DAMPs such as IL-1β and HMGB1 have the potential to activate either inflammasome or IFN-1, depending on the trigger. Autophagy has an anti-inflammatory effect by preventing inflammasome activation by two mechanisms: One mechanism involves the removal of damaged mitochondria by autophagy, while the second mechanism involves DAMPs being secreted by the secretory pathway [45,117]. Autophagy has been implicated in the regulation of chemokines such as CSF-1 and CCL2, which are involved in which function to attract monocytes and accumulate tumor-associated macrophages [5]. The protective mechanisms induced by the TRAP-induced NETs include the activation of the cytokine and protease cycles that result in the M2 or immunosuppressive macrophage and DC transformation while linking tumor autophagy and immune evasion through neutrophils [51,124]. The findings from this study provide therapeutic strategies that can help treat the immune resistance observed in TNBC. The blockage of tumor-cell autophagy leads to greater production of DAMP and IFN-I, which results in the activation and recruitment of antigen-presenting myeloid cells [45]. The combination of myeloid autophagy and LAP targeting will enable researchers to transform TAMs and DCs into new functional states that will enhance cross-priming and cytotoxic T cell responses [5,116]. Suppressing autophagy-mediated NET formation might be used for preventing neutrophil-dependent immune suppression and metastasis [51,124,125]. Such effects are probably brought about by a fine-tuned, transient, and cell-selective regulation of autophagy, possibly in association with sensitizing cancer therapies, such as immunogenic and radiotherapies, or ICB-mediated ones, to achieve therapeutic benefit without causing systemic immunosuppression [120].

### 7.4. Preclinical Data Combining Autophagy Inhibitors with Immunotherapy

*Rationale and Mechanistic Background:* The rationale for targeting autophagy to enhance anti-tumor immunity is supported by two distinct but complementary observations: (i) autophagy in cancer cells is involved in ICD-mediated pathways that enhance ATP/”find-me” signal secretion and dendritic cell activation, and (ii) tumor- and stromal-autophagy can generate immunosuppressive signals in the microenvironment (release of autophagosome-derived extracellular vesicles) that decrease T-cell activity [101,127,128]. These mechanistic findings led to the hypothesis that, in certain situations, blocking autophagy through drugs or genetic methods would make tumors more susceptible to destruction by cytotoxic lymphocytes while also enhancing the effectiveness of immune checkpoint blockade (ICB) treatment [22,27,101,129].

*Key Mechanistic Preclinical Findings:* Michaud and colleagues first showed that pre-mortem autophagy was required for ATP secretion during ICD, a process that recruits and matures antigen-presenting cells [101]. Martins et al. subsequently examined the lysosomal-PANX1-LAMP1 mechanisms that underlie autophagy-dependent ATP release [127]. By contrast, TRAPs were shown to polarize macrophages into PD-L1-high M2-like cells and to induce other suppressive myeloid programs, suggesting that tumor autophagy can also actively suppress antitumor immunity. These mechanistic studies explain why the modulation of autophagy can have opposing effects on the immune response depending on the context, timing, and the specific autophagy nodes targeted [128]. These findings align with a context-dependent framework in which therapy-induced autophagy may either enhance immunogenic signaling or reinforce immune resistance, depending on whether autophagy is occurring in tumor cells or immune compartments and whether its net effect is cytoprotective or cytotoxic.

*Proof-of-Concept Pharmacology:* PPT1 and Vps34 Inhibitors: Several preclinical studies demonstrate that chemical inhibition of specific late-stage autophagy effectors can convert ‘cold’ tumors into ‘inflamed’ and ICB-responsive states. Sharma et al. (2020) showed that the inhibition of palmitoyl-protein thioesterase 1, the target of the lysosomal inhibitors such as chloroquine and its analogs, can enhance the efficacy of the anti-PD-1 treatment in mouse melanoma models, showing reduced tumor growth and improved survival when combined with PPT1 inhibition [27]. The clinical-stage PPT1 inhibitor GNS561 (ezurpimtrostat) has analogous preclinical activity and entered first-in-human studies, underscoring translational potential [130]. Independently, selective inhibition of the class-III PI3K Vps34 (such as SAR405 and related compounds) has been shown to reprogram tumor myeloid compartments and make resistant tumors responsive to anti-PD-1/PD-L1 therapy in preclinical models. These data collectively suggest that selective inhibition of autophagy machinery components (PPT1, VPS34) may be more effective in promoting immunologically favorable TME remodeling [22,131]. However, systemic modulation of autophagy presents a major translational challenge, as inhibiting autophagy in tumor cells may enhance anti-tumor immunity but can simultaneously impair immune cell function within the tumor microenvironment.

*TNBC-Focused Preclinical Data:* TNBC has special relevance because (i) autophagy defects are common in TNBC and (ii) PD-L1-negative or immune-desert TNBC subsets are profoundly “cold” to ICB. Li et al. (2020) demonstrated that autophagy deficiency in TNBC cells caused accumulation of Tenascin-C, which protected tumor cells against T-cell-mediated cytotoxicity; genetic or pharmacological approaches that restored Tenascin-C degradation resensitized autophagy-impaired TNBC to anti-PD-1/PD-L1 therapy [100]. Additional evidence is provided by nanoparticle-based studies. Cheng et al. (2022) loaded chloroquine, chemotherapy, and immunoadjuvants plus an anti-PD-L1 antibody into multifunctional nanoparticles and observed marked tumor control, increased CD4+/CD8+ infiltrates, and prevention of lung metastasis in mouse breast cancer models (including 4T1-based TNBC models), indicating that combining autophagy blockade with checkpoint blockade can be synergistic in breast cancer preclinical systems [129]. While these results are encouraging, they also highlight the importance of selecting the appropriate autophagy node (a Molecular regulator/checkpoint in the autophagy pathway, such as TNC targets, PPT1, or VPS34) and delivery strategy for TNBC [129].

*Conflicting Preclinical Signals and Context Dependence:* Not all preclinical data uniformly favor combination therapy. Krueger et al. (2021) reported that HCQ at clinically relevant dosing impaired the therapeutic benefit of anti-PD-1 in a B16-PD-L1 melanoma model by inhibiting expansion of PD-1+TCF1+ progenitor CD8+ T cells; a result that cautions against generalizing CQ/HCQ combinations with ICB without careful dose/timing optimization [132]. In contrast, Starobinets et al. (2016) found that, in their models, systemic antimalarial autophagy inhibition did not blunt adaptive antitumor immunity, supporting the possibility of safe combination in specific settings [133]. These apparently discordant findings emphasize the importance of which autophagy inhibitor is used (non-specific lysosomotropic CQ/HCQ versus more selective PPT1/Vps34 inhibitors), the tumor model and antigenicity, and the scheduling relative to chemotherapy/ICB. Thus, preclinical results must be interpreted in a nuanced, model-by-model fashion [133].

*Practical Lessons For Translational Design:* From preclinical work several practical points emerge for TNBC-directed trials combining autophagy blockade with immunotherapy: (1) choose mechanistically rational targets (PPT1, VPS34) rather than relying solely on CQ/HCQ; (2) biomarker-guided selection (such as Tenascin-C levels, LC3/p62, baseline TILs and ICD readouts) could identify tumors most likely to benefit [100]; (3) co-delivery strategies (nanoparticles, neo-adjuvant scheduling) may enhance tumor localization and reduce systemic immune off-target effects; and (4) preclinical dose-time mapping is critical because autophagy plays cell-type-specific roles in dendritic cells, T cells and myeloid cells. Collectively, preclinical data support cautious clinical evaluation of autophagy-targeting strategies, as their effects are highly context-dependent and may vary across tumor and immune compartments. [129]. A key challenge for clinical translation is the lack of cell-type-specific targeting, as systemic autophagy modulation may produce opposing effects in tumor and immune cells.

Therefore, preclinical studies provide a substantial, though heterogeneous, body of evidence that selective autophagy inhibition can remodel the TME and enhance ICB efficacy in multiple models, including breast cancer/TNBC [100,101,127,129,131]. The balance of pro-immune and immune-suppressive autophagy functions depends on the molecular node targeted, tumor immunogenicity, and scheduling; hence, the critical need for selective inhibitors, predictive biomarkers (such as Tenascin-C, TIL phenotype, ICD markers), and careful translational pharmacology when moving from mouse to patient (Figure 5) [27,100,101,129,130,132,133,134].

## 8. Key Molecular Pathways Linking Autophagy and Resistance in TNBC

### 8.1. PI3K/AKT/mTOR Signaling

The phosphoinositide 3-kinase (PI3K)/AKT/mechanistic target of rapamycin (mTOR) signaling pathway is a master regulator of cellular growth, metabolism, and survival, and its activity has a profound effect on the autophagy machinery in TNBC [35]. This signaling pathway coordinates mitogenic and nutrient signals from outside the cell with the cell’s internal energy state to decide between anabolic growth and catabolic processes such as autophagy [36]. Canonical activation begins when receptor tyrosine kinases (RTKs) such as epidermal growth factor receptor (EGFR) or insulin-like growth factor-1 receptor (IGF-1R) undergo ligand-induced dimerization and autophosphorylation, creating phosphotyrosine docking sites for the SH2 domains of the p85 regulatory subunit of class I PI3Ks [135]. Class I PI3Ks contain a catalytic p110 subunit (α, β, or δ isoform) and a p85 regulatory subunit. Class I PI3Ks phosphorylate phosphatidylinositol 4,5 bisphosphate (PIP2) to produce phosphatidylinositol 3,4,5 trisphosphate (PIP3) on the inner surface of the plasma membrane [36]. PIP3 is a membrane anchor for pleckstrin homology domain-containing proteins, such as AKT/protein kinase B and phosphoinositide-dependent kinase-1 (PDK1), bringing these proteins into close proximity to one another for the phosphorylation events required to activate AKT [35]. PDK1 phosphorylates AKT at threonine-308, while mTOR complex 2 (mTORC2) phosphorylates serine-473, resulting in full activation of AKT kinase activity [135]. Activated AKT phosphorylates and inhibits the tuberous sclerosis complex (TSC1/TSC2), a GTPase-activating protein (GAP) for the small GTPase Rheb, thereby allowing Rheb to accumulate in its GTP-bound state and directly activate mTOR complex 1 (mTORC1) [61]. In TNBC, genetic alterations such as activating PIK3CA mutations, PTEN inactivation, and AKT isoform amplification result in the hyperactivation of the pathway, placing the cells in a ‘growth on/autophagy off’ state even under basal conditions [36,136].

mTORC1 is a multi-protein kinase complex containing mTOR, Raptor, mLST8, PRAS40, and DEPTOR, and it localizes to the cytosolic surface of lysosomes, where it senses amino acid sufficiency through the Rag GTPases and the Ragulator-v-ATPase complex [137]. When amino acids are plentiful, active Rag heterodimers recruit mTORC1 to the lysosome. mTORC1’s kinase activity is activated by Rheb-GTP. mTORC1 phosphorylates ULK1 at serine-757. This disrupts its interaction with AMPK and thus prevents the initiation of autophagy [61]. mTORC1 also phosphorylates transcription factor EB (TFEB), sequestering it in the cytoplasm and thereby repressing lysosomal biogenesis and autophagy gene expression [138]. Under energy stress, AMPK phosphorylates ULK1 at the activating sites serine-317 and serine-777 and also phosphorylates TSC2 and Raptor to inhibit mTORC1, thus releasing the autophagy blockage [35,61]. TFEB dephosphorylation allows nuclear translocation and transcription of CLEAR (coordinated lysosomal expression and regulation) genes, expanding the autophagy-lysosome system [25]. This nutrient-lysosome-signaling interface is reflected in the ability of PI3K/AKT/mTOR inhibitors, as well as chemotherapeutic agents that lower the cell’s ATP levels, to reactivate autophagy through the activation of the complex involving the kinase ULK1 and the transcription factor TFEB, which regulates lysosomal function [26,36].

The autophagy machinery downstream of the PI3K/AKT/mTOR pathway is organized into functional modules. The ULK1-ATG13-FIP200-ATG101 complex is involved in the initiation of phagophore formation, and the BECLIN 1 (BECN1)-VPS34/class III PI3K-ATG14 complex is involved in the nucleation of the isolation membrane [139]. AKT can phosphorylate BECLIN-1 at serine-295, favoring its association with 14-3-3 proteins and decreasing the activity of the VPS34 lipid kinase, which negatively regulates the nucleation of the autophagosome [140]. mTORC1-dependent suppression of TFEB reduces lysosomal hydrolases and membrane proteins needed for autophagosome maturation [138]. As elongation of autophagosomes proceeds, microtubule-associated protein 1 light chain 3 (LC3) lipidation, also called LC3-II, takes place, whereas p62/sequestosome-1 (SQSTM1) interacts with ubiquitinated targets in a process required for selective degradation [29]. As p62 is degraded by autophagy, the rate of p62 degradation is used to measure the autophagic flux (Table 2). The functional significance of p62 is that it binds to Kelch-like ECH-associated protein 1 (KEAP1) to stabilize the antioxidant protein nuclear factor erythroid 2-related factor 2 (NRF2), thus linking autophagy to antioxidant responses [29,35].

Anthracyclines and taxanes cause DNA damage, ROS accumulation, and endoplasmic reticulum stress in TNBC to activate AMPK and suppress mTORC1 activity to induce autophagy [61,139]. Survival-induced autophagy protects cancer cells by removing damaged mitochondria by mitophagy, maintains mitochondrial membrane potential, and suppresses cytochrome c release to decrease caspase activity and apoptosis [26,29]. The consequence of this is the maintenance of ATP and amino acid levels via lysosomal recycling, redox balance, and dampening of intrinsic apoptosis, thereby supporting a drug-tolerant persistent state [26,36].

Feedback loops impede therapeutic targeting. Inhibition of mTORC1 also reverses S6 kinase (S6K)-mediated suppression of insulin receptor substrate 1 (IRS1), thereby enhancing upstream PI3K/AKT signaling [150]. mTORC2 may continue phosphorylating AKT at serine 473 following selective inhibition of mTORC1, which preserves survival signals and inhibits induction of autophagy [35]. Furthermore, bypassing the RAS/RAF/MEK/ERK pathway may also lead to the phosphorylation of autophagy regulators as well as apoptosis-related proteins, thus fine-tuning the survival-death balance [135]. This may explain why monotherapy with PI3K/AKT/mTOR inhibitors is cytostatic rather than cytotoxic to TNBC cells [26,36].

The immunologic component is also linked in this regard. AKT/mTOR signaling activates PD-L1 expression via NF-κB and STAT3, leading to a suppression of T cell functions (Hoxhaj & Manning, 2020) [36]. Autophagy can also degrade MHC-1, leading to a suppression of antigen presentation [10]. It can also clear cytosolic DNA and damaged mitochondria, dampening cGAS-STING signaling and type I interferon production, thereby limiting innate immune activation [29]. This suppression of innate immune sensing can blunt the recruitment and activation of cytotoxic lymphocytes, thereby fostering an immune-cold TME that is less responsive to checkpoint blockade [10,26]. In TNBC, where immune infiltration is a key prognostic factor, the balance between PI3K/AKT/mTOR activity and autophagic flux becomes a decisive determinant of whether tumor-immune interactions are stimulatory or suppressive [29,36].

From a therapeutic standpoint, the dual role of the PI3K/AKT/mTOR axis as both a driver of resistance and a trigger of compensatory autophagy has sparked interest in combination strategies. Preclinical studies have shown that pairing PI3K inhibitors such as alpelisib, AKT inhibitors such as capivasertib, or catalytic mTOR inhibitors with autophagy blockers (such as hydroxychloroquine, ULK1 inhibitors, or VPS34 inhibitors) can convert cytostatic responses into cytotoxic ones by disrupting the metabolic scaffolding that autophagy provides under stress [26,61]. In these models, pharmacodynamic markers such as decreased phospho-S6 and phospho-4EBP1 confirm mTORC1 inhibition, while changes in ULK1 phosphorylation, TFEB nuclear translocation, LC3-II accumulation, and p62 turnover verify modulation of autophagy [35,138]. The timing and sequencing of such combinations are critical. Continuous co-administration can enhance toxicity in normal tissues that depend on basal levels of autophagy, whereas intermittent schedules of PI3K/AKT/mTOR inhibition and autophagy blockade can selectively target tumor cells at the peak of their catabolic recycling dependency [26]. The immunotherapy paradigm requires a more intricate approach, where transient PI3K/AKT/mTOR inhibition has the potential to reduce signaling and reduce PD-L1 levels, but if checkpoint blockade is initiated immediately without any attempt to reduce the autophagy surge, antigen presentation, and cGAS-STING signaling have the potential to be down-regulated [10,29].

Another component of effective translation is the selection of patients. Tumors harboring losses in PTEN, PIK3CA activating mutations, phospho-AKT, or mTORC1 hyperactivity, such as phospho-S6 positivity, have a high probability of having a strong pathway addiction and a typical autophagy rebound effect following treatment stress [36,136]. The autophagy proficiency level, defined by the presence of LC3 puncta, p62, and TFEB/TFE3, may potentially determine the level of induced autophagy following treatment [25,139]. Adaptive resistance may be evaluated by the level of reactivated phospho-AKT, ERK, and metabolic profiles, as these adaptive responses may either restore the growth on/auto-off state or establish a chronic survival state dependent on autophagy, which is susceptible to proximal autophagy inhibitors [135,150].

Thus, in summary, the PI3K/AKT/mTOR pathway is not just a linear growth signal; rather, it is a dynamic rheostat that switches autophagy either on or off in response to nutrient availability, stress signals, as well as pharmacological interventions [35,36]. In TNBC, its dysregulation underlies both chemoresistance and immune evasion, while its inhibition paradoxically licenses a survival autophagy program that sustains residual disease. Combinatorial therapeutic strategies involving pathway-specific inhibition with titrated modulation of autophagy, driven by molecular and functional biomarkers, can potentially bypass drug resistance and improve survival for this aggressive breast cancer subtype [10,26].

### 8.2. AMPK–ULK1 Axis in Autophagy Initiation and Therapeutic Resistance in TNBC

The adenosine monophosphate-activated protein kinase (AMPK)-UNC-51-like kinase 1 (ULK1) pathway is the major signaling pathway involved in the regulation of autophagy, a process of cellular homeostasis involving the degradative functions of the lysosome [66]. AMPK is a heterotrimeric serine/threonine kinase composed of a catalytic α subunit and regulatory β and γ subunits, activated when the AMP/ATP or ADP/ATP ratio rises, typically via phosphorylation of AMPKα at Thr172 by upstream liver kinase B1 (LKB1) [151]. ULK1 is a serine/threonine kinase that forms a complex with ATG13, FIP200 (FAK family-interacting protein of 200 kDa), and ATG101, initiating phagophore formation, the precursor membrane structure of the autophagosome [152,153]. In TNBCs, where metabolic stress is ubiquitous due to high rates of proliferation, hypoxia, and therapeutic stress, the AMPK–ULK1 signaling pathway represents a finely regulated survival mechanism that allows cancer cells to modulate autophagy for chemoresistance and immune escape [154].

Under nutrient-rich conditions, mTORC1 phosphorylates ULK1 at Ser758 (human numbering; Ser757 in murine ULK1), disrupting ULK1-AMPK binding and inhibiting ULK1 kinase activity, thereby preventing autophagy initiation [35,61,155]. When amino acid levels decrease, mTORC1 activity is suppressed, relieving this inhibitory phosphorylation and allowing AMPK to interact with ULK1. In the canonical model, AMPK phosphorylates ULK1 at sites such as Ser555 (human) or Ser556 (murine), activating ULK1 and triggering downstream recruitment of the class III PI3K complex composed of VPS34, BECN1, ATG14, and PIK3R4 to generate PI3P at the phagophore assembly site [60,156]. This PI3P-enriched compartment then recruits effector proteins such as WIPI2 and DFCP1, which aid in elongating the membrane via the ATG8 conjugation machinery [12]. In TNBC, this nutrient-sensing pathway is typically hijacked to maintain autophagy even when nutrients are replete, supporting anabolic growth and survival under pressure from therapy [157].

Recent work has revealed that AMPK is not always a positive regulator of ULK1. In fact, in critical situations of energy depletion caused by mitochondrial dysfunction, glucose starvation, and chemotherapy at high doses, the activation of AMPK by LKB1 has been shown to inhibit the activity of ULK1 [158,159]. In this revised paradigm, AMPK maintains a stable interaction with ULK1, preserving ULK1’s structural integrity and preventing caspase-mediated degradation, while simultaneously preventing its activation to avoid the ATP-intensive process of autophagosome biogenesis [160]. This prevents cells from worsening the energy crisis by triggering autophagy when ATP levels are significantly depleted, which is especially important in TNBC, where drugs such as doxorubicin or paclitaxel can trigger severe mitochondrial stress [154]. Once energy levels partially recover, between chemotherapy cycles, ULK1 can be rapidly reactivated from its preserved state, enabling efficient clearance of damaged organelles and macromolecules, thereby contributing to chemoresistance [82].

The logic of phosphorylation sites is central to understanding AMPK–ULK1 regulation. Phosphorylation mediated by mTORC1 at Ser758 suppresses ULK1-AMPK binding, whereas phosphorylation mediated by AMPK at Ser555 generally enhances ULK1 activation in response to moderate stress [61,155]. However, under extreme energy deficit, AMPK’s phosphorylation pattern and sustained binding can “lock” ULK1 in an inactive state despite mTORC1 inhibition [151]. This duality explains why pharmacological AMPK activators such as metformin or AICAR do not uniformly induce autophagy in TNBC cells; their effects depend on nutrient availability, energy charge, and stress duration [154]. In the immune resistance setting, this gating mechanism could temporarily downregulate autophagy-dependent antigen processing and DAMP secretion during acute immune attack, thereby reducing the tumor’s immunogenicity. Upon reactivation, the ULK1 activity could selectively eliminate immune effector-induced damage, thereby facilitating immune evasion [82,158].

Therapeutically, the AMPK–ULK1 pathway is also a multiple-point target for intervention. Inhibition of ULK1 in the post-crisis recovery phase may be able to silence autophagy-mediated repair and metabolic repletion, rendering TNBC cells susceptible to chemotherapy or immunotherapy [157,161]. Sustaining AMPK activation in energy crises could inhibit autophagy initiation and increase cytotoxicity if ULK1 protection inadvertently causes subsequent rebound survival [159]. Focusing on the interaction between AMPK and ULK1 at the plasma membrane to prevent their protective interaction without causing ULK1 to break down is another strategy that might help avoid the restart of autophagy during treatment-related stress [60,158]. Notably, mTORC1 inhibitors might not be enough to start autophagy when energy levels are low, as they are often blocked by AMPK, highlighting the need for combined approaches that consider both nutrient and energy conditions [35,155].

In TNBC, the AMPK–ULK1 pathway acts as a switch, integrating signals from nutrient sensors, energy sensors, and stress response systems to control autophagy. This process has a direct effect on how cancer cells resist chemotherapy and the immune system [82,154,158]. The fact that its control activation under moderate stress and restraint under severe energy deficit enables tumor cells to survive immediate damage and subsequently clear this damage through autophagy contributes to its strength against treatment [82,154]. Mapping ULK1 and AMPK phosphorylation states alongside metabolic profiling will be essential for designing precision strategies that disable this survival circuitry in TNBC [154].

### 8.3. YAP1–TEAD Transcriptional Regulation of Autophagy and Resistance in TNBC

The Hippo signaling pathway is a master regulator of tissue homeostasis, organ development, and tumorigenesis, and its dysregulation has significant implications for autophagy-dependent TNBC resistance. At the center of this program is YAP1, a transcriptional coactivator that lacks intrinsic DNA-binding activity and hence depends on TEA domain transcription factors (TEAD1-4) to implement its transcriptional programs [142,143,162]. In canonical Hippo-active states, the MST1/2 kinases phosphorylate and activate the LATS1/2 that phosphorylate YAP1, causing its cytoplasmic sequestration by 14-3-3 proteins, followed by proteasomal degradation [142,162]. YAP1 enters the nucleus after Hippo signaling repression ends via three pathways: oncogenic signals, mechanical stresses, and cytoskeletal changes. YAP1 interacts with TEADs to activate transcriptional programs that preferentially use enhancers to control cell growth and survival and metabolic processes [144,163]. These enhancer programs are not limited to growth control but extend to autophagy regulation, as YAP1–TEAD activity intersects with nutrient-sensing pathways and lysosomal biogenesis [164,165,166].

Mechanistically, YAP1–TEAD signaling has been shown to regulate autophagy at multiple levels. YAP1–TEAD signaling has been shown to increase biosynthetic load, leading to AMPK activation during nutrient starvation, as well as mTORC1 inhibition, leading to the removal of repression on ULK1 complexes, thereby initiating autophagy [167,168]. YAP1–TEAD also cooperates with MiT/TFE transcription factors such as TFEB to expand lysosomal capacity, ensuring efficient autophagic flux [164,165]. This crosstalk between TGFβ/SMAD signaling and epithelial–mesenchymal transition (EMT) increases basal autophagy, which in turn confers protection to TNBC cells from apoptosis and immune cell-mediated cytotoxicity [142,143]. Taken together, these mechanisms make YAP1–TEAD a transcriptional node that maintains autophagy-supported chemoresistance and immune evasion [144,145].

Therapeutically, the YAP1–TEAD complex is druggable through its dependence on TEAD autopalmitoylation. This process is a post-translational modification that is necessary for the stability and activity of TEAD, and covalent inhibitors that compete with the palmitoylation pocket destabilize TEAD, inhibit YAP1–TEAD-driven transcription, and consequently inhibit autophagy-supported resilience [169,170]. In addition, verteporfin has also shown its ability to inhibit the interaction between YAP and TEAD and its effects on oncogenic activity mediated by YAP, which includes autophagy-mediated chemoresistance, although its off-targeting effects are problematic [171,172]. Upstream normalization of Hippo kinases or mechanical signals, by targeting RhoA-ROCK, integrin-FAK, or the actin cytoskeleton, can reduce the movement of YAP1 into the nucleus and alter the enhancer landscapes, which in turn reduces the effectiveness of autophagy as a survival mechanism [169,173,174].

The immune interface once again emphasizes the YAP1-autophagy axis. YAP1 suppresses innate antiviral responses by inhibiting IRF3 dimerization and nuclear entry, whereas YAP phosphorylation by IKKε leads to its degradation in lysosomes, thereby associating the activation of innate immunity with YAP autophagy. The YAP1–TEAD activity is known to sustain the TME through the suppression of antigen presentation, the regulation of cytokine secretion, and the induction of stromal remodeling, which results in the suppression of antitumor immunity [175,176,177,178]. By targeting YAP1–TEAD, it is possible to both reduce chemoresistance that is driven by autophagy and change the immunosuppressive TME into one that supports an immune response [144,145].

### 8.4. Noncoding RNAs in TNBC: lncRNA and microRNA Regulation of Autophagy and Therapeutic Resistance

Noncoding RNAs (ncRNAs) are functional RNA species that help in the regulation of gene expression at the transcription level, as well as the post-transcription and epigenetic levels. Long noncoding RNAs (lncRNAs) are defined as ncRNAs longer than 200 nucleotides and act as a scaffold for chromatin modifiers, decoys for transcription factors, guides for the functioning of ribonucleoprotein complexes, or as sponge molecules for microRNAs in ceRNA networks [147,148,179]. MiRNAs, ~22 nucleotides in length, are processed by Drosha and Dicer and repress target mRNAs through seed-sequence pairing [147,179]. Collectively, lncRNAs and miRNAs regulate the initiation of autophagy (ULK1/2-ATG13-FIP200), nucleation of the autophagosome (VPS34-Beclin1), elongation of the autophagosome (ATG5-ATG12-ATG16L, LC3 lipidation), and the fusion of lysosomes with autophagosomes (Rab7, SNAREs), adapting the TNBC cell survival response to chemotherapy and immunotherapy [12,149,180].

LncRNAs control autophagy through direct protein interactions and ceRNA-mediated transcriptome regulation. HOTAIR facilitates the recruitment of E2F1 to autophagy gene promoters [181]. GAS5 and MALAT1 play a role in the regulation of autophagy by scaffolding or sponging autophagy-suppressive microRNAs, while PVT1 promotes chemoresistance by enhancing the stability of expression of autophagy-related genes [182,183].In TNBC, such ceRNA networks with lncRNAs are often involved in the sequestration of the miRNAs, which otherwise repress the process of autophagy and hence derepress the expression of BECN1, ATG5, ATG7, and ULK1, which maintain the cytoprotective flux [149,184,185]. All these processes are closely related to the process of EMT-like transcriptional states and metabolic changes, such as glycolysis and lipid metabolism, which are intimately [164,167,186].

Moreover, miRNAs regulate autophagy directly by controlling the mRNAs of autophagy-related genes or upstream nutrient sensors. The miR-21, miR-34a, miR-155, and miR-30 families regulate the expression of ULK1, Beclin-1, or ATG genes, suppressing or stimulating autophagy fluxes, albeit in a network-specific manner [187,188,189,190]. Given that the expression of miRNA is tightly regulated by stress-induced transcription factors, TNBC cells frequently exhibit specific miRNA expression patterns that can promote autophagy-associated resistance. Significantly, miRNA expression is known to interact with other forms of RCD. For example, the regulation of ferritinophagy and lipid peroxidation by miRNA can influence ferroptosis, especially in the context of p53-mutant TNBC [191,192,193].

From a clinical perspective, the ncRNA autophagy axes provide biomarkers and programmable targets for intervention. The autophagy reliance signatures of lncRNA may predict and classify TNBC patients for autophagy inhibition therapy, whereas the signatures of miRNA may inform us whether autophagy is cytoprotective and whether it can be inhibited [149,185]. Therapeutically, lncRNA antisense oligonucleotides may counteract the sponging effect of a ceRNA, thereby restoring the miRNA-repression balance of autophagy genes. miRNA inhibitors/mimics may restore the balance of autophagy flux, but this is a challenge due to issues of delivery and off-target effects [148,184]. Because autophagy is modular, treatments are possible. Small changes in ULK1 activation, Beclin 1 complex stability, LC3 lipidation, or lysosomal acidification can cause changes in drug tolerance and immune responsiveness [12,180]. Within TNBC’s immune-evading microenvironment, most microRNAs that regulate antigen presentation or potentiate immune suppressive cytokine production concurrently regulate autophagy, and targeting such microRNA nodes and autophagy pathways could re-educate these cancers to become immunologically relevant while also diminishing chemoresistance [176,186,192].

In summary, ncRNAs, both lncRNAs and miRNAs, constitute a dense regulatory layer that integrates autophagy with chemoresistance and immune evasion in TNBC. ncRNAs maintain the metabolic adaptation and cytoprotective flux that are necessary for cell survival through various mechanisms, including scaffolding chromatin regulators, miRNA sponging, or directly repressing autophagy genes [12,52,147,148,149,179,180,181,182,183,184,185,186,194]. The interactions of non-coding RNAs with apoptosis, ferroptosis, and immune signaling highlight their role as key communication hubs in resistance networks [187,188,189,190,191,192,193]. Hence, not only are ncRNAs biomarkers of reliance on autophagy, but they also offer therapeutic opportunities to overcome resistance to cancer cell treatment through autophagy [176].

### 8.5. Mutant vs. Wild-Type p53: Divergent Regulation of Autophagy and Therapy Response in TNBC

The tumor suppressor p53 is a master regulator of cellular stress responses, and its status (wild type versus mutant) and nuclear versus cytoplasmic localization critically determine how autophagy is wired into chemoresistance and immune evasion in TNBC. Nuclear p53 transcriptionally activates autophagy by upregulating genes that suppress mTOR signaling, i.e., TSC2, AMPK subunits, and damage-regulated autophagy modulator (DRAM), thus linking growth arrest with the capacity for degradation [195,196]. In contrast, cytoplasmic p53 suppresses basal autophagy, and its depletion paradoxically triggers autophagy, suggesting a “brake” function under homeostatic conditions [97]. This regulation is influenced by factors such as phosphorylation, acetylation, and ubiquitination, as well as stress and nutritional status, which determine whether such autophagic activity is pro-death or cytoprotective under treatment [73,98,146].

In TP53-mutant TNBC, mutations are extremely common that encompass DNA-contact mutants (such as Arg248, Arg273) and conformational mutants (such as Arg175), each of which possesses gain-of-function (GOF) phenotypes pirating autophagy that cause chemoresistance and metastasis [197,198,199]. The GOF mutant p53 stabilizes Beclin-1 complexes, reprograms AMPK-mTOR signaling, and modulates lysosomal biogenesis. This shifts autophagy toward a cytoprotective flux. This is accompanied by the inhibition of immunogenic cell death [37,191]. Moreover, in addition to genetic and other modifications of TP53, MDM2-mediated degradation and epigenetic silencing of TP53 play a role in decreasing the nuclear autophagy induction with variable displacement of cytoplasmic repression [107,167,200]. These are stratified according to p53 genotype and subcellular localization when administered concurrently with chemotherapy or immunotherapy in combination with autophagy modulators [73,98].

The autophagy-p53 interplay extends into the selection of the RCD pathway. Removal of damaged mitochondria by mitophagy eliminates ROS, thereby preventing apoptosis in p53-defective TNBC, and inhibition of autophagy can make cells sensitive to chemotherapy agents or induce the ferroptosis or necroptosis pathway [193,201]. Conversely, in wild-type p53 contexts, p53-driven autophagy can cooperate with apoptosis and immune activation to produce tumor-suppressive outcomes [107,146]. For combinatorial approaches of ferroptosis inducers mediated by autophagy inhibition or activation of the DRAM pathway with modulation of selective autophagy, it is critical to calibrate these effects to avoid the development of resistant clones that utilize macropinocytosis or other scavenging pathways [73,167]. Therefore, a personalized design that incorporates information on p53 mutational class, levels of expression, compartment dynamics, autophagic flux, and immune context is crucial for rationally overcoming drug resistance in TNBC (Table 2) [37,98].

**Figure 6:**
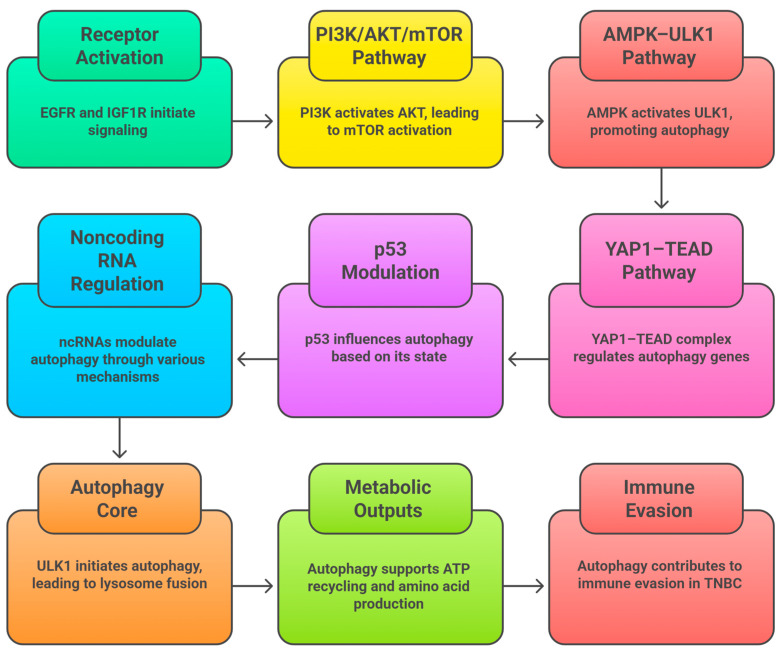
Molecular pathways linking autophagy and resistance in TNBC. The figure illustrates how various signaling pathways link autophagy to TNBC survival and resistance. Activation of receptors such as EGFR and IGF1R feeds into the PI3K/AKT/mTOR pathway, which, under nutrient-rich conditions, tends to suppress autophagy. However, in a nutrient-poor microenvironment, AMPK activates ULK1, which activates the beginning stages of autophagosome formation. Two transcriptional regulators have different effects on autophagy: YAP1–TEAD, which is constitutively active and promotes autophagy, and p53, which has a bidirectional impact on autophagy that is highly dependent on the cellular microenvironment. Non-coding microRNAs also play an important role in regulating autophagy by controlling ATG levels. Once activated, autophagosomes form membrane-bound compartments that eventually fuse with lysosomes, thereby initiating cellular breakdown. The cellular materials are recycled, thus allowing TNBC cells to survive the antimicrobial effects of chemotherapy. In addition, the continuous autophagic process leads to immune escape, resulting in a poor treatment response.

Finally, p53’s autophagy regulation converges with Hippo-YAP and ncRNA circuits. Activating AMPK is a simultaneous action to modulate p53 transcriptional programs and autophagy initiation. Finally, lncRNA, miRNA, and ceRNA establish a network that modulates the expression of p53 target genes and the autophagy machinery to establish a resistance phenotype, with coordination from enhancer reprogramming involving YAP1–TEAD [149,164,165]. This level of understanding positions p53 as a central node coupling genome surveillance with autophagy and survival, and dictates how cells within TNBC types respond to drug-induced stress and immune pressure; a promising approach to disrupt immunologically resistant clones via TEAD/ncRNA and p53/RCD targets is also a way to reactivate long-term anti-tumor control [144,145] (Figure 6).

## 9. Biomarkers of Autophagic Activity in TNBC

To improve clinical interpretability, biomarkers discussed in this section are categorized based on their functional role as prognostic, predictive, or pharmacodynamic markers. Monitoring autophagy in TNBC is essential for understanding how tumor cells adapt to chemotherapy and how the immune response evolves. Due to the dynamic nature of autophagy, caution should be taken in the interpretation of autophagy biomarkers, with a focus on flux rather than absolute measurement [180]. The most common autophagy markers used in studies include LC3B (pharmacodynamic biomarker) puncta and LC3-II/I ratio measurements. Initially, the protein LC3 was revealed as a mammalian ortholog of the Atg8 protein in yeast and was associated with the autophagosomes’ membranes upon lipidation [63]. The conversion of LC3-I to LC3-II is a hallmark of the formation of autophagosomes, and the localization of LC3B using fluorescent labeling has been used as a focal point [202]. However, under conditions of autophagy induction as well as autophagy blockade in the degradation phase of the pathway, an accumulation in levels of LC3-II has been recorded [203]. Chemotherapy and hypoxia induce high levels of LC3-II in TNBC cells, while an mCherry-GFP-LC3 system reconstitutes LC3 and stains autophagosomes as well as autolysosomes, thereby proving autophagy flux [180]. Although LC3-based assay systems are critical, their results should be confirmed with lysosomal inhibitors and other approaches [63,180,202,203].

Another critical biomarker is p62 (pharmacodynamic biomarker/SQSTM1, a selective autophagy receptor that binds ubiquitinated cargo and LC3, targeting substrates for degradation. Because p62 itself is degraded by autophagy, its abundance inversely reflects autophagic activity [69]. p62 downregulation usually points to an active process of flux, while its overexpression points to dysfunctional autophagy or an increased number of targets for degradation [167]. Yet p62 is also transcriptionally regulated by NRF2 and NF-κB, and is phosphorylated on sites such as Ser403, which affects cargo preference for binding and makes it difficult to interpret [203]. In TNBC, p62 sequestration correlates with resistance to PARP inhibition, as well as chemotherapy, and highlights its role in proteostasis and survival signaling [204]. Consequently, p62 levels should be used in conjunction with the LC3-II flux assay, with core assessment of transcription and post-transcriptional modifications to prevent misattribution [69,167,203,204].

Core autophagy machinery proteins such as Beclin-1, ATG5, and ATG7 also serve as biomarkers (primarily pharmacodynamic biomarkers). Beclin-1 regulates autophagosome nucleation by interacting with class III PI3K complexes, and its interaction with UVRAG and Rubicon enhances and inhibits autophagy, respectively ([80]). Beclin-1 gene expression is heterogeneously down-regulated in breast cancer. This implies that reduced Beclin-1 expression is a manifestation of impaired autophagy. On the contrary, the overexpression of Beclin-1 serves as an adaptive response to stress. This is related to autophagy-generated survival functions [205]. ATG5 and ATG7 proteins are critical regulators of LC3 lipidation and autophagosome elongation. ATG7 clinical studies proposed a positive association with patient survival in certain TNBC types, although it may be exploited under chemotherapy pressure to induce survival autophagy [206]. These proteins are best considered functionally, i.e., Beclin-1 with LC3 and p62 [167,180].

Circulating autophagy-related miRNAs provide a minimally invasive biomarker (predictive biomarkers of therapeutic response) that integrates systemic tumor biology. The downregulation of miR-30a derepresses autophagy and promotes resistance by directly targeting Beclin-1 [189]. Additionally, miR-34a, which is regulated by p53, also targets autophagy-related genes, whereas miR-21 and miR-155, which are elevated in TNBC patients, are associated with PI3K/AKT/mTOR and NF-κB signaling pathways, thereby indirectly influencing autophagy activity [207]. Dysregulated circulating microRNAs have been implicated as predictors of drug response in patients with TNBC, reinforcing their role as autophagy-associated markers, as they are associated with autophagy activity [208]. Panels with low levels of miR30A/miR34A and high levels of miR21/miR155 may predict resistance by autophagy when such a prediction is tested against tissue flux [189,207,208].

Imaging biomarkers (pharmacodynamic biomarkers) represent an emerging frontier. Tandem LC3 reporters allow preclinical imaging of autophagosome maturation [203]. Clinically, it is possible to detect metabolic and microenvironmental stressful conditions that induce autophagy patterns by means of FDG-PET imaging and diffusion-weighted-MRI scans; however, these are indirect measures [141,164]. PET imaging agents that specifically target the role of lysosomes and components involved in autophagy are also being developed and validated; however, it is important to notice that imaging techniques alone cannot determine if autophagy exists and that tissue-based methods are occasionally needed to validate imaging results [180]. It is recommended that imaging results are used in cooperation with biopsy-based measurements involving LC3-II and p62 for a suitable understanding of these mechanisms [167]. These imaging techniques offer a contextualization that does not substitute for molecular techniques [141,164,167,180,203].

In summary, LC3B, p62, and autophagy core components primarily serve as pharmacodynamic biomarkers of autophagy activity, whereas circulating miRNAs show promise as predictive biomarkers of drug efficacy. Conversely, existing prognostic biomarkers in TNBC, including TILs, are highlighted in other sections as immune-related rather than autophagy-associated. An orthogonal biomarker panel is composed of LC3 puncta and LC3-II/I ratios, p62/SQSTM1, Beclin-1/ATG5/ATG7, circulating miRNAs, and imaging surrogates. Each marker can give only a partial perspective, and together, TNBC autophagy sensibly can be credited with chemoresistance and immune evasion [63,69,80,180,189,202,203,206,207,208].

## 10. Therapeutic Strategies Targeting Autophagy

### 10.1. Classical Inhibitors: Chloroquine and Hydroxychloroquine

CQ and HCQ are classical autophagy inhibitors. CQ and HCQ are weak bases that accumulate within acidic vesicles, thereby increasing lysosomal pH and hindering the fusion of autophagosomes with lysosomes [209,210]. In TNBC, where autophagy is also induced in a general manner as a survival response to chemotherapy or targeted therapy stress, CQ has been shown to restore paclitaxel sensitivity to PI3K/AKT inhibitors and increase paclitaxel efficacy, validating autophagy as a compensatory resistance pathway [74,76]. In addition to inhibiting flux, CQ/HCQ also suppresses NF-κB signaling by inhibiting autophagic degradation of p47 and, in turn, suppressing pro-survival inflammatory signaling [26,211]. There have been few Phase II clinical trials with HCQ and chemotherapy in human breast cancer, although retinopathy and myelosuppression as dose-limiting toxicities make pharmacodynamic studies necessary in this class of drug [210,212]. The most developed autophagy inhibitors are CQ and HCQ, which have both shown chemosensitizing activities in TNBC patients, although their effects on lysosomal structures and general toxicities necessitate biomarker-based dosing strategies [26,76,209,211].

### 10.2. Next-Generation Small Molecules

VPS34 and ATG4B Inhibitors: Second-generation autophagy inhibitors, on the other hand, target autophagy upstream regulators directly. In autophagy, class III PI3K, also known as VPS34, plays a role in the production of PI3P, which initiates nucleation of the autophagosome membrane. The role of VPS34 inhibition in TNBC cell autophagy initiation is essential in rendering cells sensitive to nutrient starvation and chemotherapy drugs [213,214]. However, the major role of class III PI3K also includes regulation of endosomal trafficking. The concern mainly arises from the side effects [215,216]. Medicinal chemistry design aims at optimizing kinome selectivity and targeted delivery to tumor tissues to reduce systemic effects [174,216]. ATG4B, a cysteine protease involved in the LC3/GABARAP maturation, is a second target of interest; inhibition causes accumulation of immature autophagosomes and flux-defective autophagy with increased chemosensitivity in TNBC xenografts [217,218]. Structural studies have found small molecules that specifically bind to ATG4B’s catalytic pocket. However, achieving effective bioavailability and pharmacokinetics is still a challenge [218,219]. VPS34 and ATG4B inhibitor agents represent a level of pathway-specific suppression of autophagy with greater mechanism-based specificity than the CQ/HCQ approach, but their applicability will depend upon the delivery of agents to tumor cells, as well as reducing the toxicity associated with vesicular trafficking [214,215,216,218].

### 10.3. Natural Compounds: Tanshinone I and Curcumin Derivatives

Natural compounds are found to manage autophagy through a multi-target approach to targets, including stress kinases, ROS, and transcription. On the contrary, Tanshinone I, a compound obtained from Salvia miltiorrhiza, targets the autophagy observed at the late stage in TNBC by inhibiting AKT/p38 MAPK and thereby sensitizing doxorubicin [220,221]. Further studies indicate that tanshinones are capable of inducing autophagy as well as inhibiting it, thus necessitating flux analysis to determine whether cytoprotective or cytotoxic autophagy [222,223]. Curcumin and its derivatives modulate autophagy through PI3K/AKT/mTOR and NF-κB pathways, often inducing apoptosis when combined with autophagy inhibitors [224,225]. In TNBC, curcumin derivatives have been optimized for stability and bioavailability, showing synergistic effects with chemotherapy by tipping the balance toward apoptotic rather than protective autophagy [225,226]. Tanshinone I and curcumin derivatives promote chemosensitization by targeting autophagy, in addition to modulating immunometabolism. Nonetheless, mechanistic validation of these compounds, in terms of their dual roles, is essential [220,222,224,225].

### 10.4. Gene-Silencing Approaches: siRNA and CRISPR

Gene silencing technologies allow therapeutic targeting of autophagy regulators and determine how TNBC develops resistance through new mechanisms. In particular, siRNA targeting ATG5, ATG7, and BECN1 reduces autophagy levels and renders TNBC cells sensitive to chemotherapy. These approaches are delivered with lipid nanoparticles and conjugation approaches that use a ligand [227,228]. CRISPR-Cas9 genome-wide screens have identified autophagy-related weaknesses such as ATG4B and ULK1. This shows they can be targeted for drugs [229,230]. Knocking out ATG4B in TNBC using CRISPR also renders the cancer more sensitive to DNA-damaging agents. These results indicate that ATG4B plays a role in chemoresistance [231,232]. siRNA enables targeted reduction in autophagy drivers. CRISPR identifies resistance networks. Together, they improve therapy for autophagy-targeted TNBC [227,229,230,231].

### 10.5. Combination Regimens with Chemotherapy, Targeted Agents, and Immunotherapies

Inhibition of autophagy is optimal when used with stress-inducing therapies, which make cells dependent on autophagy. TNBC combination treatment with CQ/PI3KAKT inhibitors and paclitaxel was superior to single-agent therapies for tumor control in TNBC patients, thereby confirming the role of autophagy as a survival compensatory mechanism [74,76]. Sequential treatment with inducers and inhibitors of autophagy, including mTOR inhibitors and CQ, respectively, can induce cytotoxic autophagic stress, but timing is critical to avoid induction of cytoprotective effects [26,233]. Clinically, everolimus plus platinum chemotherapy has shown improved outcomes in advanced TNBC, highlighting the metabolic autophagy interface [234,235]. Other combinational immunotherapies are also promising, and autophagy inhibition would increase antigen presentation and the recycling of immunosuppressive cells, thereby enhancing the efficacy of checkpoint blockade immunotherapy [236,237,238]. Neoadjuvant chemoimmunotherapy trials in TNBC suggest that modulation of autophagy could further enhance T-cell priming and tumor clearance [28,238].

Combinations rationally designed, especially with chemotherapy, PI3K/AKT inhibitors, and immunotherapy, stand for the most interesting application of autophagy inhibition in TNBC, assuming a biomarker-driven approach will be adopted for the purpose [28,76,233,234].

## 11. Preclinical and Clinical Studies

### 11.1. Preclinical Evidence in TNBC (In Vitro and In Vivo Studies)

This section primarily summarizes evidence derived from preclinical TNBC models, although selected findings from other tumor types are included where TNBC-specific data are limited. Inhibition of autophagy resensitizes EGFR-driven TNBC cells to tyrosine kinase inhibitors. In tests with TNBC cells, combining an autophagy inhibitor with gefitinib induced mitochondrial-mediated cell death, thereby reducing tumor growth more effectively than gefitinib alone. This was reported by Liu et al. (2017) in PLOS ONE [239]. The way it works is that blocking autophagy increases the likelihood that cells will undergo apoptosis. This fits with the idea that autophagy helps cells survive stress and become resistant to drugs, as seen in earlier studies [239]. This research gives clear proof that blocking autophagy at a late stage can reverse resistance to targeted therapy in TNBC models [239]. Blocking a late-stage autophagy pathway resulted in an increase in chemotherapy efficacy, where toosendanin, a lysosomal flux inhibitor, sensitized TNBC to irinotecan by enhancing DNA damage and apoptosis, and also blocked autophagy, as reported by Zhang et al. (2022) [240]. Notably, the exposure levels that caused LC3-II accumulation and p62/SQSTM1 retention were enhanced by irinotecan-induced cytotoxicity. This supports the idea that blocking flux is the mechanism. [240]. In vivo, the effective doses did not show any overt toxicity signals in the mouse models, so it is plausible for combination chemotherapy [240].

Synergy between autophagy inhibitors and pathway-targeted drugs has been demonstrated in TNBC. Chloroquine blocked adaptive resistance to PI3K/AKT inhibitors and increased antitumor activity when combined with paclitaxel in various cell lines and mouse models, leading to lower tumor burden and delayed tumor regrowth, as reported by Cocco and colleagues [76]. Hence, the findings proved the survival role of autophagy upon PI3K/AKT inhibitor treatment. Also, the study has proven the potential of hydroxychloroquine analogs as chemosensitizers. It has revealed the possibility of re-establishing chemosensitization by the combination of flux inhibitors with pathway-targeted drugs, as demonstrated in TNBC [76]. Attenuation of enhanced immunity stromal suppression by autophagy provides a strong rationale for a combination approach, as autophagy suppression, along with anlotinib and PD-1 inhibitors, enhanced cancer-associated fibroblast functions and tumor suppressor pathways in NSCLC models. These findings are derived from non-TNBC models and should be interpreted with caution when extrapolated to TNBC. These findings set a precedent for NSCLC, which is transferable to TNBC’s TME despite TNBC’s heterogeneity as a disease [241]. Since tumors differ, inhibiting them and boosting antigen presentation and T-cell function are viable treatment approaches, particularly for chemo-immunotherapy. Adaptive autophagy might reduce immunogenicity. This cross-indication evidence supports combining autophagy blockade with immunotherapy or antiangiogenics to remodel the TME [241].

The autophagy modulation in TNBC is bidirectional, with suppression of autophagy and induction of anti-tumor activities via AMPK/mTOR/ULK1 pathway signaling. Icariin induced autophagy-related cell stress, inhibiting TNBC cell growth in vitro and in xenograft models in a context-dependent manner. In addition, the induction may be cytotoxic under certain conditions, whereas resistance contexts are more likely to be associated with inhibitory modes of sensitization. In that case, the stage of the signaling pathway is crucial in determining the time [82]. Together, these observations highlight that autophagy regulatory modality must be regulated according to TNBC stress biology as well as to therapeutic intent [82]. Enhanced immune stroma suppression by autophagy provides a rationale for the combination approach, which was supported by the study where inhibition with anlotinib, PD-1 blockade, and autophagy inhibition reprogrammed the functional activities and tumor suppressive pathways among cancer-associated fibroblasts, which provided a precedent for the counterpart in the TNBC microenvironment, despite the heterogeneity of the condition [241]. Such a model will provide the rationale for the capacity of CQ/HCQ analogs to exploit the potential for synergistic activity with DNA damage and microtubule-acting agents in preclinical models of TNBC, in which adaptive autophagy, induced by cytotoxic stress, is a survival mechanism [76,233]. Pre-clinical evidence supports the intermittent administration pattern of PK-based profiling in relation to peaks [233].

### 11.2. Clinical Translational Landscape and Gaps

This section includes both indirect clinical evidence and emerging translational approaches related to autophagy in TNBC, highlighting current gaps in autophagy-specific clinical trials. Even though the number of clinical entries of autophagy-specific agents in TNBC treatment is small, the continuum of resistance reversal programs includes mechanisms in which the autophagy inhibitors are involved, and online sources are available for access and information on active and completed TNBC research trials, across different combinations of chemotherapy and immunotherapy, and other oncology treatment options [242,243]. These resources allow for filtering out studies involving agents that modulate autophagy, such as CQ/HCQ, and those exploring endpoints for autophagy biomarkers (LC3-II, p62), although autophagy might not be a primary target [242,243]. From this perspective, there is a disconnect between preclinical robust synergy and autophagy-targeting TNBC clinical trials [242].

Ongoing clinical trials are examining the role of autophagy in TNBC treatment. Specifically, a Phase 1 clinical trial (NCT06347068) is evaluating autologous iC9-CAR.B7-H3 cells in TNBC patients who have relapsed or are resistant to treatment. This trial will provide insight into how immune reprogramming occurs within the tumor, since autophagy may affect antigen processing and T-cell stimulation [244]. Another ongoing study involving Sacituzumab tirumotecan in combination with pembrolizumab and compared with the choice of therapy for the physician in a TNBC population with no pathological complete responses (NCT06393374) is a testament to the effect of autophagy in chemoresistance and the role of the pathway in autonomous survival in the face of cytotoxic agents [245]. Even though these experiments do not directly involve the use of an autophagy inhibitor, there is still a biological connection that warrants the study of autophagy [244,245].

**Figure 7:**
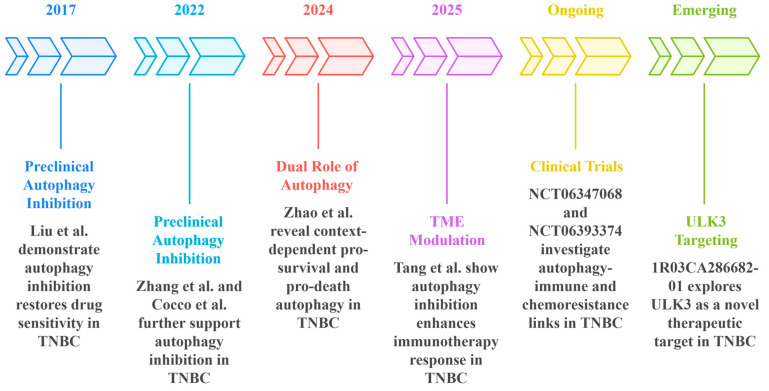
Timeline of preclinical and clinical advances in the targeting of autophagy in TNBC. The following figure shows how some of the major points from early mechanistic work to new translational approaches have been connected. Early preclinical studies from 2017 found that autophagy blockade re-sensitized EGFR-positive TNBC models to targeted therapies, identifying autophagy’s role in drug resistance. More preclinical studies from 2022 confirmed that advanced autophagy suppression improved the efficacy of chemotherapy and PI3K/AKT signaling pathway inhibitors. In 2024, research focused on the context-dependent dual role of autophagy, with a focus on its survival and death-promoting effects on tumor cell growth. In 2025, the focus was on “Modulation of TME”, with autophagy inhibition highlighted as a strategy to improve immunotherapy. With clinical trials ongoing to determine various anti-cancer effects of immunotherapy, and new prospects emerging with ULK3 inhibitors, it is clear that there are growing opportunities in the field of autophagy-directed approaches in TNBC [76,82,239,240,241,244,245,246].

Upstream pipelining signals predict upcoming autophagy-axis trials, attempts to manipulate ULK3 in TNBC (Project 1R03CA286682 01) indicate early-stage research interest in targeting autophagy pathways [246]. As novel late-stage autophagy inhibitors move from initial concepts to early safety testing, trials for TNBC listed on the NCT registry ought to start including autophagy measurements or combination treatments (National Institutes of Health, n.d.; ClinicalTrials.gov, n.d) [242,246]. The finding aligns with earlier studies that showed the medicine works on PI3K/AKT and irinotecan treatments [76,240] (Figure 7).

### 11.3. Clinical Limitations and Lack of TNBC-Specific Autophagy Trials

Despite strong preclinical evidence, clinical trials specifically targeting autophagy in TNBC remain limited. The profile of toxicity depends on the phase of the pathway and the lysosomotropic activities. The toxic profile of the CQ/HCQ flux inhibitor class has already been established, including retinopathy and QT prolongation, which require dose adjustments when used with cytotoxic and target therapies, as briefly discussed in the complete review [79]. Based on the application of CQ in TNBC models, efficacy has improved without a cost-prohibitive toxicity profile at the doses used. However, patients need to be monitored when applying CQ, including cardiac and ophthalmologic monitoring and assessment of drug interactions, as highlighted in the literature [76,79]. The schedules for combination therapies require careful consideration of efficacy and cumulative risk. From the PK viewpoint, the lysosomotropic compounds have a high capacity for tissue distribution and lysosomal sequestration. This implies that the compounds can be retained in cells and interact with pathways that require an acidic environment. The PK profile of the compound supports periodic administration and the coincidental timing of the late-autophagy inhibitor with the peaks of chemotherapy-induced stress, enabling conversion to apoptosis without toxicity [79,233]. Biomarker-based timing based on LC3-II and p62/SQSTM1 levels may be used to confirm flux inhibition within the optimal therapeutic window [79].

Most available clinical studies involve combination therapies in which autophagy modulation is not the primary endpoint. For the novel late-stage inhibitors such as toosendanin, it has been shown in mouse models that chemosensitization and flux inhibition are effective, and the toxicity is acceptable at efficacious doses, though the PK/PD in humans and off-target toxicity are not yet tested and will require stepwise phase I evaluation [240]. This approach should take into consideration exposure-response relationships, lysosomal engagement, and TME immunologic activity in consideration of the role of autophagy in antigen processing and CAF biology [240,241]. This approach should help elucidate the therapeutic window in chemo-IO combinations, as Tang et al. (2025) describe [241]. For combination regimens, dose identification is also a necessity. For example, with PI3K/AKT inhibitors, autophagy inhibition can result in decreased survival signaling and possibly increased metabolic stress. Titration dosing is suggested, with staggered dosing to avoid overlapping toxicities, as described in Cocco et al. (2022) and Hassan et al. (2024) [76,79]. PK dosing strategy for late-stage inhibitors following peak chemotherapy with an ‘induce then block’ approach links with the mechanisms and apoptosis conversion, as suggested by Hassan et al. (2024) and Liu et al. (2020) [79,233]. These approaches, by the way, were based on preclinical synergies among TNBC [76].

The safety profile of immuno-oncology compounds will need to be assessed and modulated to address immune-related adverse events on T cells that have the potential for rebalancing stromal inhibition and antigen processing through the suppression of autophagy that modulates immune cell homeostasis [241]. In addition, cytokine profiles, T cell function, and CAF markers must be incorporated in the Phase I studies to define limits for combination regimens with PD-1/PDL1 inhibitors [241]. This is particularly important in the context of TNBC chemotherapy and immunotherapy, where modulating autophagy can improve or alter responses [241]. In TNBC models, late-stage autophagy inhibition has been shown to resensitize tumors to targeted therapies and chemotherapies, such as EGFR TKIs, irinotecan, and PI3K/AKT inhibitors in vitro and in vivo [76,239,240]. Registries of clinical trials reflect few autophagy-specific TNBC programs but increasing related activity (CAR-T, chemo-IO), where autophagy biology is highly applicable, with upstream pipeline indicators (ULK3) of imminent autophagy-specific trials [242,243,246]. Toxicity and PK profiles define lysosomotropic concentration, QT/retinal toxicity, and immune modulation to favor biomarker-driven, PK-coordinated “induce-then-block” regimens to shift adaptive autophagy into apoptotic commitment without sacrificing tolerability [79,233,241].

## 12. Challenges and Considerations

This part struggles with the paradoxes and functional limitations that govern autophagy-directed strategies for reversing chemoresistance and immune resistance in TNBC. The objective here is to provide an overview of why this field requires accurate mechanistic design, stratification, and delivery strategies to modulate autophagy in cancer compartments in a manner that is tightly regulated without compromising host defense and tissue integrity.

*Dual Role of Autophagy in Cancer: Protumor Versus Antitumor:* The dual nature of autophagy is due to the rewiring of autophagy in different stages, contexts, and stress conditions, where it has the potential to restrict tumorigenic processes by limiting genomic instability and proteotoxic stress, and it can also consolidate pre-existing tumors by promoting metabolic plasticity, hypoxia tolerance, and drug resistance, especially in poor-N environments of TNBC [247,248,249]. Mechanistically, catabolic flux provides redox balance, quality control, and bioenergetic adaptation during chemotherapy stress while maintaining cell viability of drug-resistant clones; loss of autophagy triggers a cascade of events leading to impaired cell clearance and initiation of inflammatory responses, which in turn determine the benefits or damage of the treatment, as a function of initiation, timing, and stage of tumor development [247,248]. CSCs seem to show a paradox in that autophagy, when induced, supports CSC maintenance and stemness during cytotoxic stress; conversely, in other cases, its induction restrains oncogenic signals, leading to a dilemma for a general inhibition approach [92].

Therapeutically, it will be important to place inhibition in the context of stress from the microenvironment and lineage dependencies; in the context of glycolytic dependence and hypoxia as a primary stress in TNBC, it is likely that autophagy will play a role as a chemotherapy survival buffer, indicating a therapeutic time frame during which temporary inhibition may resensitize the tumor at a very low likelihood of long-term suppression leading to pro-inflammatory, protumor activities [247,248]. The accuracy of the switch will be measured by utilizing stress signaling and flux biomarkers, which will help in identifying when the switch for autophagy has occurred in favor of supporting tumor survival rather than tumor suppression, thus avoiding absolute treatments that may result in compensatory resistance [92,249]. Autophagy can function in limiting initial tumorigenesis, including therapy-resistant TNBC, in providing a buffer against stress from metabolism and oxidation, and the therapeutic strategy may be crucial in modulating autophagy in relation to tumor development, CSC, and tumor microenvironmental stressors [92,247,248,249].

*Impact on Host Immunity and Normal Tissues:* Autophagy interacts with innate and adaptive immunity across processes, including antigen processing and presentation, MHC class II presentation, inflammasomes, and pathogen clearance. Therefore, it is systemic inhibition of autophagy is expected to result in defects in antigen presentation, dendritic cell priming, and macrophage function [250,251]. In cancer, autophagy can support immune escape via degradation of cytotoxic factors and restoration of the secretome, but in immune cells, autophagy is required for homeostasis, suppressing ectopic inflammation, and creating a dichotomy where, in the context of cancer, suppression of tumor-directed autophagy can paradoxically interfere with immune function [251,252]. For normal cells, especially for post-mitotic cells like neurons and cardiomyocytes, as well as for rapidly dividing cells like epithelial cells, autophagy is important for organelle quality control and proteostasis. Systemic disruption of autophagy may cause neurotoxicity, cardiomyopathy, hepatic steatosis, or increased susceptibility to infection [252,253]. In relation to the combination of immunotherapy, this has a dual effect: inhibiting autophagy will enhance the signals for immunogenic cell death and antigen availability, while inhibiting antigen-presenting cells will abrogate the effect of the checkpoint inhibitors [251,254]. Therefore, the design of the compound for clinical utility must aim to have compartmental selectivity to minimize the effect on the immune tissue while maintaining the basal flux in healthy tissue, using the tumor-specific stress to trigger the payload; this balance is crucial to avoid the effect on the immune system [250,253]. Modulation of autophagy affects antigen presentation, innate immunity, and tissue proteostasis; the design of a compound for cancer treatment must avoid the suppressive effects on immune system priming and cytotoxicity to autophagy-dependent normal tissues, especially when combined with immunotherapy [250,251,252,253,254].

*Patient Stratification and Predictive Biomarkers:* This heterogeneity in the dependence on autophagy in TNBC necessitates stratification based on dynamic flux, stress pathway signatures, and circulating/ctDNA biomarkers. Expression of LC3/Beclin1 is not adequate without assessing the functionality of autophagosome clearance and lysosomal activity [255]. The measurement of autophagy flux (e.g., LC3-II turnover in lysosomal inhibitor media), dynamic assessment of p62/SQSTM1 levels, and proteolytic activity of lysosomes could be complemented with hypoxia signatures, mTOR/AMPK pathway activation status, and CSC signatures for diagnosing tumors that would benefit from anti-autophagy treatment [256]. The newly emerging liquid tumor bio-markers that hold promise for application in anti-cancer therapy are non-invasive predictors of chemoradiotherapy resistance and include extracellular vesicle miR-30c signatures and autophagy phenotypes of circulating tumor cells [257]. From the methodological point of view, the integration of multi-omics data with the help of machine learning can improve the accuracy of patient segmentation if the models leverage flux data instead of static data for isolated markers, and the models are trained on clinically annotated data with therapy response endpoints [258]. The predictive panel will need to integrate autophagy flux markers, stress pathway activation (PI3K-AKT-mTOR/AMPK), microenvironmental hypoxia/acidosis markers, and immune competence markers to predict the benefit of modulation of autophagy in combination with chemo- and immunotherapy [255,256]. The integration of multi-omics data based on the use of the machine learning-validated platform for the identification of responders will require robust markers of autophagy flux, stress pathway activation, and microenvironmental markers, along with liquid biopsy markers such as EV miRNAs and autophagic CTC phenotypes [255,256,257,258].

*Drug Delivery and Tumor Specificity:* As autophagy is essential for normal tissue and immune function, the delivery platforms must be designed to promote tumor specificity, for example, using TME-responsive nanocarriers, which utilize the low pH, high ROS, and hypoxia, and actively target the tumor cells to minimize modulation in tumor tissues [81,259]. pH-sensitive nanoparticles, redox-sensitive nanoparticles, and ligand-targeting nanocarriers, such as EGFR, EGFRvIII, and integrins, for the delivery of autophagy inhibitors and theranostic agents targeting the PI3K-AKT-mTOR signaling pathways have also shown promise for improving the therapeutic ratio with minimal systemic drug exposure [81,260]. Even spatial accuracy is critical, for example, in relation to the TME, which is autophagy-dependent to a certain extent in tumor stroma and CSCs, and for which nanoparticles must be able to penetrate tissues, overcome the ECM, and escape endosomes while minimizing uptake by immune cells in lymphoid organs [259,261]. Researchers have found that when and how autophagy is suppressed can vary significantly across treatment designs. A few groups have experimented with delivery systems that hold the drug until a signal, either from the tumor itself or an external source. When this process synchronizes with chemotherapy or radiotherapy, the result appears to increase tumor stress while allowing normal autophagy to resume in the latter stage [260]. In the case of the application of immunotherapy, the formats must not suppress global autophagy in antigen-presenting cells; microenvironment-activated formats can be designed to confine the action to the TME, and “smart” formats can be designed to integrate autophagy modulation with immunostimulatory domains to “make the tumor more visible” [259,261]. Drug delivery formats with the potential for selective targeting, dosage with flux calibration, and combination scheduling will be needed to outsmart the dual nature of autophagy with safety potential in the clinic for the treatment of TNBC [81,260]. Tumor-selective nanomedicines responsive to the TME and targeting the TNBC microenvironment can confine the action of autophagy modulation, allow transient and schedule-synchronized inhibition, reduce the impact on the immune and normal tissues, and improve the potential for combination with chemo- and immunotherapy [81,259,260,261].

## 13. Future Directions

*Biomarker-Driven Trial Design:* Trials for TNBC to be conducted in the near future should be biomarker-driven, and stratification must be performed based on signatures of autophagy flux and resistance-related transcriptional programs. Single-cell transcriptomics have shown that resistant subclones of TNBC co-activate autophagy, lipid metabolism, and PD-L1 stabilization, and are expected to be useful as predictive biomarkers for adaptive trial arms. Regulon analysis also suggests that enhancer rewiring and ATG-related regulons associated with chemoresistance could serve as biomarkers for patient selection. Of special interest are parameters related to the TME, such as TAM polarization status, cytokine profiles, and stromal autophagy induction, which should be incorporated into trial designs, as they play a significant role in chemoresistance and immune evasion. The inclusion of such biomarkers will enable adaptation of trial arms according to real-time pharmacodynamics related to autophagy flux, such as levels of LC3-II and p62, to increase the chances of therapeutic responses [230,262]. When planning TNBC trials, one promising approach is to stratify patients by key molecular or cellular biomarkers. By examining clonal transcriptomic patterns alongside regulatory signaling and TME features, researchers can adjust treatments as the disease evolves. This type of adaptive design could facilitate the counteracting of resistance that develops through autophagy [263,264,265].

*Nanoparticle and Targeted Delivery Systems:* Nanoparticle-based delivery systems offer a powerful means to selectively modulate autophagy in TNBC. Some research groups have taken a more “engineered” approach, designing nanoparticles that deliver inhibitors of the PI3K/AKT/mTOR pathway or the lysosomal pathway. The ability of these nanoparticles to deliver their cargo in an acidic environment has the potential to enable spatial and temporal targeting of the therapy towards cancer cells, while healthy cells remain “safe” [81,259]. Mannose or scavenger receptor ligand-conjugated nanoparticles targeting TAM have the potential of simultaneous repolarization of macrophages towards the M1 phenotype, suppression of autophagy induced by the TME, and induction of anti-tumor immunity [265,266]. At the same time, mRNA nanoparticle-mediated platforms have the potential for the transient knockdown of the autophagy genes ATG5 or ATG7 in a cell-type-specific manner without the need for permanent genomic modification in patient-derived xenograft models [267]. The design of nanoparticles incorporating pH-responsive release systems, endosomal escape motifs, and flux-reporting cargo has the potential to enhance therapeutic precision, enabling real-time in vivo monitoring of autophagy modulation [81,268]. In addition to this, biomimetic coatings, such as platelet membranes or ligands targeted at integrins, can optimize the accumulation of drugs in tumors while reducing clearance by the reticuloendothelial system, thereby optimizing pharmacokinetics and payload delivery [259,268]. These approaches describe how nanomedicine can inactivate autophagy-dependent resistance networks through the co-delivery of synergistic payloads, including autophagy inhibitors with ferroptosis inducers or immune checkpoint modulators [262,267]. Autophagy-mediated nanoparticles that are engineered to cross the TME, engage with the lysosomes, and be implicated in immune-cell-specific delivery are a translationally viable strategy to overcome chemoresistance and immune evasion for TNBC [81,266,268].

*Synthetic Lethality Screens to Identify Synergistic Partners:* Synthetic lethality approaches enable systematic identification of vulnerabilities that emerge during autophagy inhibition. Genome-wide CRISPR-Cas9 screens in TNBC have previously identified resistant subtypes and can also be applied to autophagy-modulated conditions to reveal potential collaborators in DNA repair, ferroptosis, and lysosomal biogenesis [230,269]. PARP inhibition in BRCA-defective TNBC, for example, might be complemented by the concurrent inhibition of autophagy, while ferroptosis inducers might act on lipid peroxidation weaknesses that become revealed under autophagy inhibition [262,270]. Computational algorithms, including SLAYER, integrate dependency maps with multi-omics data to identify synthetic lethal pairs relevant to the clinic and narrow down the target space to drugs with sufficient therapeutic windows [271]. Interestingly enough, these screens must be conducted under conditions of stress, such as hypoxia, nutrient deprivation, or co-culture with the immune system, in order to justify context-dependent killing pertinent to drug-resistant TME [265,272]. Screening by negative selection can also uncover liabilities related to endolysosomal trafficking and mitophagy regulation and predict combinations of autophagy inhibitors and inducers of lethal ROS bursts, or inhibitors of lysosomal biogenesis [81,259]. Synthetic lethality programs, integrated with autophagy-modulated states and TME context, can identify druggable partnerships, especially in DNA repair, ferroptosis, and lysosomal biogenesis, which collapse survival redundancies in TNBC [269,270,271].

*Integrating Computational Modeling and Systems Biology:* Computational modeling and systems biology tools are essential for predicting interactions between autophagy, chemoresistance, and immune evasion. Multi-scale models incorporating single-cell transcriptomics, proteostasis quantification, and spatial immunophenotyping can model tipping points in which autophagy inhibition synergizes with ferroptosis induction or checkpoint blockade [267,272]. Virtual twin models of TNBC tumors, derived from patient datasets, can iteratively optimize synthetic lethal partners and dosing regimens [230,271]. These models will also need to consider immune-metabolic coupling, i.e., the overlap of lipid rewiring, PD-L1 stability, and autophagy, to optimize therapy sequencing [262,265]. The addition of nanoparticle pharmacokinetics and intracellular trafficking to these models will enable the prediction of payload localization within autophagic compartments, thereby informing delivery strategies with biomarker thresholds [81,268]. These combination system approaches have the potential to de-risk combination treatments and individualize autophagy-targeting regimens in TNBC [263,264]. Systems biology and computational modeling in clonal dynamics, regulatory networks, immune-metabolic coupling, and nanodelivery kinetics can de-risk therapeutic decisions and tailor autophagy-targeted regimens for TNBC [81,264,271].

## 14. Conclusions

Autophagy plays a key, context-dependent role in the biology of TNBC: it functions as both a protector of proteostasis and genomic integrity in normal cells and an adaptive survival system that TNBCs hijack to withstand cytotoxic and immune-mediated pressure [13,29]. At the mechanistic level, stress-induced ULK1-Beclin1-VPS34 signaling and downstream LC3 lipidation enable TNBC cells to cycle macromolecules, maintain mitochondrial quality, and mitigate pro-inflammatory danger signaling features that underlie chemoresistance and immune evasion [12,21].

This review has demonstrated that increased autophagic flux is correlated with diminished cGAS-STING signaling, decreased antigen presentation, stabilization of immunosuppressive ECM components, including Tenascin-C, and maintenance of quiescence among cancer stem cells. Therefore, autophagy serves as an overarching axis that connects cell-intrinsic chemotolerance with tumor-extrinsic immune suppression in TNBC [10,86,237].

There is preclinical evidence supporting the therapeutic rationale for inhibiting autophagy to resensitize chemotherapy- and immunotherapy-refractory TNBC to these therapies, but it also highlights a critical context-dependent effect. Late-end lysosomotropic agents (chloroquine/hydroxychloroquine) and early-node inhibitors (ULK1/VPS34) each modify tumor biology distinctively: flux blockade often results in the enhancement of genotoxin-induced DNA damage and apoptotic commitment, but not always through the complete inhibition of chemotactic gradients (e.g., CXCL10) and thereby potentially enhance T-cell infiltration in high-flux tumors [45,74]. Significantly, studies with genome-altering and pharmacological agents demonstrate that the overall anti-tumor outcome depends on baseline flux, tumor subtypes, and immune competency variables that tip the balance in favor of benefit within other TNBC subsets but risk undesirable immune suppression and compensatory adaptations within other TNBC subsets [41,45].

The translational question is thus not whether to target autophagy, which is increasingly justified, but how, when, and whom. Temporally accurate strategies guided by biomarkers are critical: real-time readouts of autophagic flux (turnover of LC3-II and p62), quantitation of cytosolic dsDNA and cGAS-STING signaling, TIL/clonal TCR measures, and TME characteristics like TNC expression and lactate/lactylation signatures need to be combined to forecast therapeutic windows where transient, tumor-preferring inhibition of autophagy turns its cytoprotective flux against the tumor and renders it susceptible to the immune response [28,262]. Adaptive clinical trials employing pharmacodynamic endpoints to modulate or escalate combinations of genotoxins and autophagy conditioners ± PD-1/PD-L1 blockade will be key to mitigating toxicity and optimizing immunogenicity and cell death [230,263].

Delivery and selectivity are comparable. Since systemic, long-standing autophagy inhibition has the potential to compromise APC function, host immunity, and tissue homeostasis, the next-generation strategies should highlight tumor-targeted nanocarriers, TME-responsive cargos, and transient “induce-then-block” time programming to localize modulation spatiotemporally to malignant compartments [259,260]. Such delivery platforms can co-deliver autophagy inhibitors and chemotherapy/immune adjuvants and are anticipated to enhance the therapeutic index by sparing immune function and normal tissues while targeting resistant subclones within the tumor and CSC niches that survive through autophagy [81,261].

Some practical research priorities follow directly from the evidence reviewed herein. First, prospective validation of multiplexed biomarkers (flux markers, cGAS-STING readouts, TNC, lactate signatures, and single-cell transcriptional states) is required to stratify patients for autophagy-modulation arms [180,264]. Second, mechanistic mapping of where autophagy intersects with DDR, PD-L1 trafficking, and secretory autophagy within defined subtypes of TNBC will define the most promising synthetic lethal partners (i.e., ATR/CHK1, ferroptosis inducers, lysosomal biogenesis inhibitors) and rational triplet combinations [265,270]. Third, stringently designed windows of opportunity and neoadjuvant trials with inclusion of strong pharmacodynamics and immune profiling can test hypotheses regarding scheduling (short-pulse inhibition during ICD induction versus chronic blockade) and quantify downstream consequences on antigen presentation and T-cell priming [6,101].

Finally, selective inhibition of autophagy represents an integral, biologically based strategy for overcoming the twin obstacles of chemoresistance and immunoresistance in TNBC. Success is less a matter of bulk inhibition than of selectivity. Converting autophagy from a tumor protective mechanism to therapeutic liability requires (i) the construction of multi-faceted biomarkers to determine dependency and timing, (ii) the application of select delivery and transient temporal control to ensure preservation of host immunity, and (iii) the calibration of combination regimens to exploit synthetic lethality and immunogenicity to overcome compensatory escape. With these principles firmly in place, the field can translate promising preclinical data into durable clinical benefits in patients with TNBC and thereby turn an extensively deployed survival mechanism into an exploitable liability to treat one of the most challenging in breast oncology [26,28,29]. It is now clear that autophagy has a role in TNBC, either directly within tumors or indirectly via immune modulation, depending on the specific context. Nevertheless, many questions remain about the types of cells involved in autophagy regulation, the timing of autophagy, and its relationship with immunotherapy. Thus, future clinical trials must focus on selective autophagy targeting, biomarker-driven patient stratification, and assessment of the dual impact of autophagy in both tumor and immune contexts.

## Figures and Tables

**Table 1:** Functional impact of autophagy on therapeutic resistance and immune evasion in TNBC. Summary of autophagy-related pathways implicated in resistance to therapy and immune escape in TNBC. Includes mechanisms linked to metabolic changes (nutrient limitation, stress conditions), with associated shifts in redox balance. EMT; DNA damage and repair in the context of treatment response. Effects on antitumor immunity were noted in some cases, mainly where autophagy intersects with immune signaling. Pharmacological modulators listed for selected pathways. Overall role varies by context; in some settings, supporting tumor survival, in others, influencing response to combination therapy.

Impact on TNBC Resistance and Immune Evasion	Representative Modulators/Drugs
Suppresses autophagy and promotes metabolic growth, chemoresistance, and PD-L1-mediated immune evasion [18,19,48,54]	Alpelisib, Capivasertib, Everolimus, Temsirolimus
Maintains energy homeostasis and enables survival under chemotherapy-induced stress [16,83]	Metformin, AICAR, SBI-0206965
Enhances autophagosome formation and promotes chemoresistance [29,45]	SAR405, BH3 mimetics
Sustains redox balance and prevents apoptosis [58,84]	Hydroxychloroquine, Bafilomycin A1
Increases lysosomal capacity and metabolic adaptability [74,78]	Trehalose, Lys05
Promotes EMT, metabolic plasticity, and immune suppression [44,88]	Verteporfin, TEAD inhibitors
Mutant p53-driven autophagy enhances chemoresistance and immune evasion [97,98]	Nutlin-3, Cisplatin, Doxorubicin
Fine-tunes cytoprotective autophagy and drug tolerance [99]	miRNA mimics/inhibitors
Reduces ROS and suppresses cGAS-STING signaling, enabling immune escape [85]	CCCP, Parkin activators
Decreases antigen presentation and limits immunotherapy response [10,100]	Pembrolizumab, Atezolizumab

**Table 2:** Major molecular pathways that control autophagy in TNBC. The following table presents the major signaling pathways and molecular factors involved in autophagy regulation in TNBC, classified by their function within the autophagy pathway and mode of action. The first column lists the main molecular upstream regulators, transcriptional regulators, and molecules controlling selective autophagy. Column two identifies all these autophagy regulators based on the functions they play in the autophagy process: induction, formation of the autophagosome, cargo selection, degradation, lysosomal biogenesis, and regulation of the whole process. Column three presents the basic molecular mechanisms underlying their effects on autophagy.

Pathway/Molecule	Functional Role in Autophagy	Mechanism/Interaction
PI3K/AKT/mTORC1 Axis [18,19,35,36]	Central regulator of cell growth and nutrient signaling; negatively controls autophagy	PI3K activation promotes AKT phosphorylation, which activates mTORC1. mTORC1 phosphorylates ULK1 (Ser757), inhibiting autophagy initiation and TFEB translocation.
AMPK–ULK1 Axis [60,61,66]	Positive regulator of autophagy under metabolic or therapeutic stress	AMPK phosphorylates ULK1 at Ser555 to induce autophagy and suppresses mTORC1 via TSC2 and Raptor phosphorylation.
Beclin-1/VPS34 Complex [26,29,59,60]	Initiates autophagosome nucleation	Forms PI3P-enriched membranes; inhibited by AKT signaling or Bcl-2 binding.
LC3/p62 System [12,62,63]	Marker of autophagic flux and cargo degradation	LC3 lipidation (LC3-II) enables autophagosome maturation; p62 links ubiquitinated cargo to LC3.
TFEB/Lysosomal Biogenesis [138,141]	Transcriptional regulator of autophagy and lysosomal genes	mTORC1 phosphorylation retains TFEB in the cytoplasm; dephosphorylation enables nuclear translocation and CLEAR gene activation.
YAP1–TEAD Axis [142,143,144,145]	Transcriptional activator linking Hippo signaling and autophagy	YAP1–TEAD upregulates ATG and lysosomal genes; interacts with AMPK/mTOR pathways.
p53 (WT and Mutant) [97,98,146]	Dual regulator of autophagy	Nuclear p53 induces autophagy; cytoplasmic p53 inhibits it; mutant p53 enhances cytoprotective autophagy.
Noncoding RNAs (lncRNAs/miRNAs) [147,148,149]	Epigenetic and post-transcriptional regulators	lncRNAs act as miRNA sponges; miRNAs target ULK1, Beclin-1, ATG5 transcripts.
Mitophagy (PINK1/Parkin) [23,75]	Selective mitochondrial quality control	PINK1 recruits Parkin to damaged mitochondria, triggering LC3-mediated degradation.
Autophagy-Immune Cross-talk [10,54,85]	Interface between autophagy and immune signaling	Autophagy regulates MHC-I turnover, cytosolic DNA clearance, and PD-L1 modulation.

## Data Availability

No new data were created or analyzed in this study.
